# Modular Engineering of *Saccharomyces cerevisiae* for De Novo Biosynthesis of Genistein

**DOI:** 10.3390/microorganisms10071402

**Published:** 2022-07-12

**Authors:** Yonghui Meng, Xue Liu, Lijuan Zhang, Guang-Rong Zhao

**Affiliations:** 1 Frontiers Science Center for Synthetic Biology and Key Laboratory of Systems Bioengineering (Ministry of Education), School of Chemical Engineering and Technology, Tianjin University, Yaguan Road 135, Jinnan District, Tianjin 300350, China; mengyonghui_2019@tju.edu.cn (Y.M.); hiliuxue@tju.edu.cn (X.L.); zyljzhang@tju.edu.cn (L.Z.); 2 Georgia Tech Shenzhen Institute, Tianjin University, Dashi Road 1, Nanshan District, Shenzhen 518055, China

**Keywords:** genistein, modular engineering, *Saccharomyces cerevisiae*, metabolic engineering, synthetic biology

## Abstract

Genistein, a nutraceutical isoflavone, has various pharmaceutical and biological activities which benefit human health via soy-containing food intake. This study aimed to construct *Saccharomyces cerevisiae* to produce genistein from sugar via a modular engineering strategy. In the midstream module, various sources of chalcone synthases and chalcone isomerase-like proteins were tested which enhanced the naringenin production from *p*-coumaric acid by decreasing the formation of the byproduct. The upstream module was reshaped to enhance the metabolic flux to *p*-coumaric acid from glucose by overexpressing the genes in the tyrosine biosynthetic pathway and deleting the competing genes. The downstream module was rebuilt to produce genistein from naringenin by pairing various isoflavone synthases and cytochrome P450 reductases. The optimal pair was used for the de novo biosynthesis of genistein with a titer of 31.02 mg/L from sucrose at 25 °C. This is the first report on the de novo biosynthesis of genistein in engineered *S. cerevisiae* to date. This work shows promising potential for producing flavonoids and isoflavonoids by modular metabolic engineering.

## 1. Introduction

Genistein (5,7,4′-trihydroxyisoflavone), a natural isoflavone found mainly in leguminous plants [1], has antioxidant and estrogen-like activities, which benefit human health in cardio-protection, anti-osteoporosis, and anticancer effects via daily nutrition intake from food [2,3]. However, industrial production of genistein is facing prominent challenges due to the low abundance of legumes and the complex extraction process, inefficient chemical synthesis, and an environmentally unfriendly route [4]. Thus, the heterologous biosynthesis of isoflavonoids in microbes by synthetic biology offers great potential for the development of a green bioindustry [5].

For the past few years, the genistein biosynthetic pathway has been well-characterized in plants [6,7]. It starts from the single-step conversion of tyrosine to *p*-coumaric acid (CA) by tyrosine ammonia lyase (TAL), or the two-step conversion of phenylalanine to CA by phenylalanine ammonia lyase (PAL) and cinnamate 4-hydroxylase (C4H). CA is then esterized with CoA to *p*-coumarate-CoA by *p*-coumarate-CoA ligase (4CL). One molecule of *p*-coumarate-CoA and three molecules of malonyl-CoA are condensed to naringenin chalcone by chalcone synthase (CHS) and further isomerized to naringenin by chalcone isomerase (CHI). CHS acts as the key enzyme in the CA to naringenin synthesis pathway, and promiscuous CHS catalyzes the formation of bis-noryangonin (BNY) and *p*-coumaroyltriacetic acid lactone (CTAL) as by-products, which would be partially inhibited by the assistance of CHI-like protein (CHIL) in plant and in vitro experiments [8,9,10]. Naringenin is converted to genistein by three enzymes, including isoflavone synthase (IFS), cytochrome P450 reductase (CPR), and 2-hydroxyisoflavanone dehydratase (HID). IFS, a cytochrome P450 enzyme (CYP) with its associated CPR which shuttles the electrons from the reducing power (NADPH) to the heme iron center of CYP to activate molecular oxygen, catalyzes the aryl migration of the naringenin B-ring from C2 to C3 to generate the intermediate 2-hydroxyisoflavone, which is converted to genistein by HID [11].

Since *S. cerevisiae* is generally recognized as a safe (GRAS) organism, it has been employed to produce isoflavonoids [5,12,13]. By feeding precursor naringenin, the recombinant *S. cerevisiae* with heterologous expression of IFS produced genistein, and additional expression of plant CPR and HID could increase the production of genistein [14,15]. Expressing seven heterologous enzymes (PAL, C4H, CPR, 4CL, CHS, CHI, and IFS) in yeast synthesized a minor amount of genistein (0.1 mg/L) with supplementation of phenylalanine, and the titer of genistein was slightly increased when precursor CA or naringenin was fed [16]. Recently, daidzein, another important isoflavone, was produced from glucose in *S. cerevisiae* [13]. However, more efforts of metabolic engineering are required to produce genistein from sugar in yeast.

**Figure 1:**
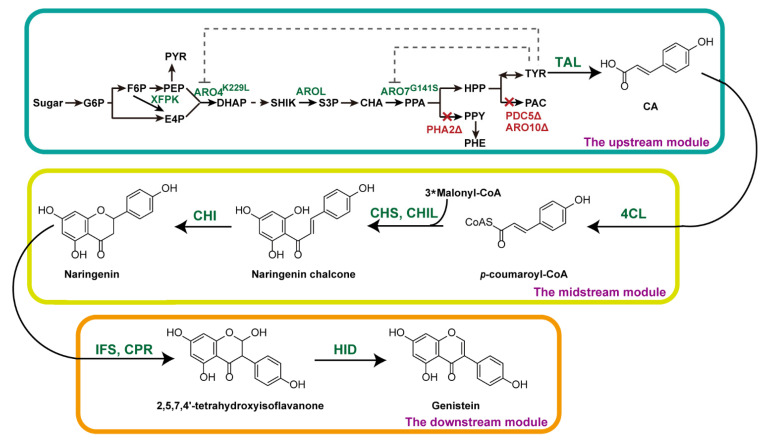
Schematic illustration of the de novo biosynthetic pathway of genistein in *S. cerevisia*e. The dashed line indicates the feedback inhibition by tyrosine. The red crosses indicate the deleted genes. XFPK, phosphoketolases; ARO4, DAHP synthase; AROL, shikimate kinase; ARO7, chorismite mutase; PHA2, prephenate dehydrogenase; PDC5, pyruvate decarboxylase; ARO10, phenylpyruvate decarboxylase; TAL, tyrosine ammonia lyase; 4CL, *p*-coumarate-CoA ligase; CHS, chalcone synthase; CHIL, chalcone isomerase-like protein; CHI, chalcone isomerase; IFS, isoflavone synthase; CPR, cytochrome P450 enzyme reductase; HID, 2-hydroxyisoflavanone dehydratase; G6P, glucose-6-phosphate; F6P, fructose-6-phosphate; PEP, phosphoenolpyruvate; PYR, pyruvate; E4P, erythrose 4-phosphate; DAHP, 3-deoxy-D-arabino-heptulosonate-7-phosphate; SHIK, shikimic acid; S3P, shikimate-3-phosphate; CHA, chorismic acid; PPA, prephenate; HPP, *p*-hydroxyphenylpyruvate; PPY, phenylpyruvate; PHE, phenylalanine; TYR, tyrosine; PAC, *p*-hydroxyacetaldehyde; CA, *p*-coumaric acid.

In this study, we divide the de novo biosynthetic pathway of genistein into three modules in *S. cerevisiae* (Figure 1). We firstly optimized the midstream module by screening biosynthetic enzymes and then localizing the pathway enzymes into the yeast organelles to improve the production of naringenin from CA. Secondly, we rewired the upstream module for the CA biosynthesis by derepressing the feedback inhibition of tyrosine and knocking out the competing branch pathways. Thirdly, we optimized the downstream module for genistein biosynthesis by combinatorial coupling of IFSs and CPRs from different plants. The metabolically engineered strain YH40 containing three modules first realized the de novo biosynthesis of genistein in yeast and improved the production after the fermentation optimization.

## 2. Materials and Methods

### 2.1. Strains, Media, and Reagents

*Escherichia coli* DH5α (Biomed, Beijing, China) was used for the construction and amplification of plasmids. Luria-Bertani (LB) medium (1% NaCl, 1% tryptone, and 0.5% yeast extract) supplemented with 100 μg/mL ampicillin or 50 μg/mL streptomycin when needed was used for *E. coli* cultivation at 37 °C.

*S. cerevisiae* CEN.PK2-1C (*MATa*; *ura*3*-*52, *trp*1*-*289, *leu*2*-*3112, *his*3*Δ*1, *MAL*2*-*8*C*, *SUC*2) was used as the starting strain for genistein production. The genomic DNA of *S. cerevisiae* BY4741 (*MATa*, *his*3*Δ*1, *leu*2*Δ*0, *met*15*Δ*0, *ura*3*Δ*0), which is derived from the originally sequenced *S. cerevisiae* S288c, was used as the template to amplify promoters and terminators by PCR. Synthetic complete (SC) drop-out medium (2% glucose, 0.67% yeast nitrogen base, and 0.2% amino acid drop-out mix) was used for the selection of auxotroph and cultivation of engineered strains at 30 °C, additionally supplemented with 10 g/L leucine, 2 g/L histidine, 2 g/L tryptophan, 2 g/L uracil, if necessary. Yeast extract peptone dextrose (YPD) medium (1% yeast extract, 2% peptone, and 2% glucose) was used for the routine yeast cultivation, the preparation of competent cells, and the loss of the Cre recombinant plasmids. All of the solid media used in this study contained 1.5% agar. To explore the favorable carbon source for the production of genistein, the final strain YH40 was cultivated in SC medium with 2% sucrose, 1% glucose-1% glycerol, or 1% sucrose-1% glycerol as alternative carbon sources, respectively.

Tyrosine, CA, naringenin, and genistein standards were purchased from Heowns Biotech Co., Ltd. (Tianjin, China). Ethyl acetate and HPLC grade of acetonitrile, methanol, and acetic acid were purchased from Concord Tech (Tianjin, China). The ClonExpress II One Step Cloning Kit was obtained from New Cell & Molecular Biotech Co., Ltd. (Suzhou, China). The DNA Polymerases of Phanta Super Fidelity and Taq were obtained from Vazyme (Nanjing, China). Yeast Genomic DNA Kit was purchased from Zoman Biotechnology Co., Ltd. (Beijing, China).

### 2.2. Construction of Plasmids and Strains

The sources of heterologous genes used in this study were listed in Table S1. Among them, the genes *FjTAL*, *Ha*4*CL*, *EbCHS*, *ErCHI*, *SbCHIL*, *PhCHIL*, *Bbxfpk*, *LjIFS*, *TpIFS*, *GmCPR*, *LjCPR*, and *GmHID* were synthesized by Tsingke Biotechnology Co., Ltd. (Beijing, China) in codon-optimized versions for *S. cerevisiae* and listed in Table S2, and other genes were synthesized in previous work [17,18]. The sequences of all promoters and terminators were listed in Table S1, which were amplified from the genomic DNA of *S. cerevisiae* BY4741 by PCR with primers listed in Table S3. All of the endogenous genes (*ARO*4, *ARO*7, *COXIV*, *PEX*3, *ACC*1) and the upstream and downstream homologous fragments of integration loci (*YPRC∆*15, *HO*, 1622*b*, 308*a*, *PHA*2, *YORW∆*17, *delta*) were amplified from the genomic DNA of CEN.PK2-1C. The *EcAROL* gene was amplified from the genomic DNA of *E. coli* DH5α. The sequence of *loxp-HIS*3*-loxp* was amplified from the plasmid pXP320 [19]. The marker genes (*TRP*1, *LEU*2, and *URA*3*)* were amplified from serial pESC plasmids (GenScript) and assembled with the *loxp* fragment by the overlap extension PCR (OE-PCR). The mutations *ARO*4*^K^*^229*L*^, *ARO*7*^G^*^141*S*^, and *ACC*1*^S^*^659*A*, *S*686*A*, *S*1157*A*^ (*ACC*1*^AAA^*) [20,21] were obtained by OE-PCR using the primers of point-directed mutagenesis. All of the PCR primers used in this study were synthesized by GENEWIZ (Suzhou, China) and listed in Table S3.

These genes were assembled into expression cassettes with the corresponding promoters and terminators by OE-PCR. For constructing expression cassettes of CHS-4CL-CHI, the three resulting fragments (P*_SED_*_1_-*CHS*-T*_ENO_*_2_ with different sources of *CHS*, P*_TEF_*_1_-*Ha*4*CL*-T*_GPM_*_1_, and P*_TEF_*_2_-*ErCHI*-T*_GPD_*) were cloned into pRS426 using ClonExpress II One-Step Cloning Kit following the manufacturer’s instructions to generate plasmids pMNG1 to pMNG8. Similarly, the fragment (P*_PGK_*_1_-*CHIL*-T*_GPD_* with different sources of *CHIL*) was cloned into pRS426 to generate plasmids pMNG12 to pMNG15. The (GGGGS)_3_ linker was used to generate fused fragments P*_TDH_*_3_-*Ha*4*CL*-(GGGGS)_3_-*EGFP-*T*_ACS_*_1_, P*_TDH_*_3_-*MLS-Ha*4*CL*-(GGGGS)_3_-*EGFP-*T*_ACS_*_1_ and P*_TDH_*_3_-*Ha*4*CL*-(GGGGS)_3_-*EGFP-ePTS*1*-*T*_ACS_*_1_ by OE-PCR, and then cloned into pRS416 to generate plasmids pMNG17, pMNG18, and pMNG19, respectively.

The gene deletion and insertion on chromosomes were performed using the yeast homologous recombination method. For complete deletion of the *PDC*5 gene, the upstream and downstream 600-bp homologous arms of *PDC*5 and marker gene *loxp-TRP*1*-loxp* were assembled into the fragment PDC5_UP_-*loxp-TRP*1*-loxp*-PDC5_DOWN_ by OE-PCR, and then transformed into strain YH27. Similarly, the *ARO*10 gene was deleted. For the integration of the pathway module, the three fragments YORW∆17_up_*-loxp-TRP*1*-loxp*-P*_TDH_*_1_-*LjIFS*-T*_PGK_*_1_, T*_PGK_*_1_-P*_PDC_*_1_-*LjCPR*-T*_TDH_*_2_, and T*_TDH_*_2*-*_P*_CCW_*_12_-*GmHID*-T*_TEF_*_1_-YORW∆17_down_ were assembled into one large fragment and then integrated into the *YORW∆*17 locus after transformation into strain YH31. Similar operations were carried out to integrate at the *YPRC∆*15, *HO*, 1622*b*, 308*a*, and *delta* loci. For the integration of the *ARO*4*^K^*^229*L*^, *ARO*7*^G^*^141*S*^, and *EcAROL* expression cassettes into the *PHA*2 locus, four fragments PHA2_up_-*loxp-LEU*2*-loxp*, P*_TDH_*_3_-*ARO*4*^K^*^229*L*^-T*_ADH_*_1_, P*_HXT_*_7_-*ARO*7*^G^*^141*S*^-T*_TEF_*_1_, and P*_HXK_*_1_-*EcAROL*-T*_CYC_*_1_-PHA2_down_ were assembled with the plasmid pCDFDuet-1 backbone into a single construct, which was amplified by PCR, and then transformed into strain YH30. Plasmids used in this study were listed in Table S4, and all plasmids and fragments were verified by sequencing before yeast transformation.

Naringenin and genistein-producing strains were constructed by transforming expression plasmids or DNA integration fragments to the corresponding host using the EX-Yeast Transformation Kit (ZOMANBIO, Beijing, China). The marker gene was removed by the *Cre-loxp* recombination system using the plasmid pCRE. The yeast clones were screened by PCR using KOD One^TM^ PCR Master Mix (Toyobo, Osaka, Japan), and then verified by DNA sequencing. All of the strains were listed in Table 1, and the pedigree of engineered yeast strains was presented in Figure S1.

### 2.3. Fermentation Conditions

For shake-flask fermentation, a single colony was picked from the YPD or SC agar plate and inoculated into 3 mL of SC medium supplemented with the corresponding amino acids and cultivated at 30 °C, 250 rpm for 20 h. Then, precultures were transferred to 5 mL of appropriate SC medium with an initial OD_600_ of 0.1 and cultivated at 30 °C, 250 rpm for 20 h. Next, the seed culture was inoculated to 50 mL of appropriate SC (containing 2% glucose, 2% sucrose, 1% glucose-1% glycerol, or 1% sucrose-1% glycerol, respectively) medium in a 250 mL shake-flask with an initial OD_600_ of 0.1, and cultivated at 30 °C, 250 rpm for 72 h. The stock solutions for CA (50 g/L) and naringenin (25 g/L) were dissolved in ethanol and DMSO, respectively, and sterile-filtered.

Fermentation experiments were performed in triplicate, and the average data are shown. Error bars represent the standard deviation of three biological replicates.

### 2.4. Biomass and Metabolite Analysis

The optical density (OD) of yeast cells was measured at 600 nm (OD_600_) by a TU-1810 spectrophotometer (Persee, Beijing, China). For CA analysis, 1 mL of fermentation broth was taken out and extracted with an equal volume of methanol. For naringenin and genistein analyses, 5 mL of ethyl acetate was added to 3 mL of fermentation broth, followed by vortex for 2 h. After centrifugation at 5400× *g* for 8 min, 3 mL of the organic layer was taken out for rotary evaporation and resuspended in 500 μL of methanol. After filtration through the filter film (0.22 μm), the samples were analyzed using a Primaide HPLC system (Hitachi, Tokyo, Japan) equipped with a UV detector. Naringenin and CA were measured at 290 nm and genistein was measured at 260 nm using a C18 column (250 mm × 4.6 mm, 5 μm, Thermo, Waltham, MA, USA) at a flow rate of 1 mL/min and temperature of 30 °C. The mobile phase for naringenin and CA detection consisted of 30% acetonitrile, 70% water, and 0.5% acetic acid, while the mobile phase for genistein detection consisted of 48% methanol, 52% water, and 0.1% formic acid. All of the HPLC analyses were quantified using a five-point calibration curve and the *R*^2^ coefficient for the calibration curve was higher than 0.99.

### 2.5. Fluorescence Microscopy

To confirm the subcellular localization of Ha4CL, organelles and Ha4CL were labeled with red fluorescent protein mCherry and green fluorescent protein EGFP, respectively. Strains YH20C, YH20M, or YH23P were cultivated in 3 mL fresh SC-His-Ura media at 30 °C at 250 rpm overnight. Precultures were transferred to 25 mL of the same media with an initial OD_600_ of 0.1 at 30 °C, 250 rpm for 60 h. Cells were collected by centrifugation and washed twice with the same volume of phosphate buffered saline (PBS) (pH 7.4), and then diluted to an OD_600_ of 0.4–0.8 with PBS. Subsequently, 2.5 μL of preparations were directly plated on the slide. The subcellular localization of Ha4CL in mitochondria and peroxisomes was observed at excitation wavelengths of 488 nm for EGFP and 585 nm for mCherry using a Nikon A1R HD25 confocal laser scanning microscope combined with a Nikon TI2-E automatically inverted microscope. The images were processed and exported by NIS-Elements AR software (Nikon, Tokyo, Japan).

## 3. Results

### 3.1. Reconstruction of the Midstream Module for Biosynthesis of Naringenin from CA

**Figure 2:**
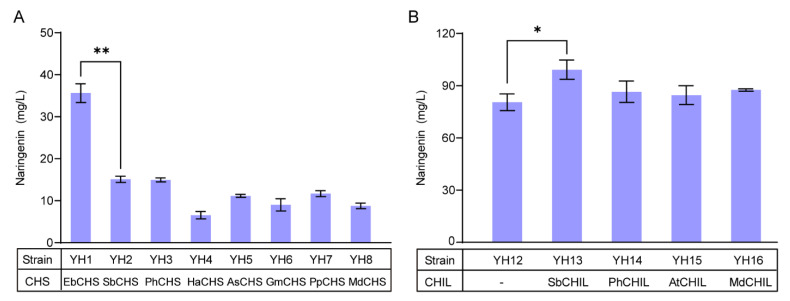
Reconstruction of the midstream module for the biosynthesis of naringenin from CA. (**A**) Screening of CHSs for naringenin production. (**B**) Screening of CHILs for naringenin production. CA (200 mg/L) was supplemented at 0 h. The data are the means and standard deviations of three replicates (*, *p* < 0.05; **, *p* < 0.01; Student’s *t*-test).

In order to obtain a more efficient CHS in yeast, we chose eight *CHS* candidates from different plants, and co-overexpressed with 4*CL* from *Helianthus annuus* (*Ha*4*CL*) and *CHI* from *Eubacterium ramulus* (*ErCHI*) in a high-copy vector pRS426, generating plasmids pMNG1 to pMNG8. After transformation into the wild-type yeast strain CEN.PK2-1C, the resulting strains YH1 to YH8 were cultivated in SC-Ura media supplemented with 200 mg/L of CA. The fermentation results showed that all eight strains produced naringenin with various titers (Figure 2A). Among them, strain YH1 expressing EbCHS produced 35.64 mg/L of naringenin, which was 240% of strains YH2 and YH3. Strains YH4 to YH8 produced less naringenin, accompanied by a large amount of CA accumulation (Figure S2), indicating that HaCHS, AsCHS, GmCHS, PpCHS, and MdCHS were less efficient in yeast. Then, we chromosomally integrated the *Ha*4*CL*, *EbCHS*, and *ErCHI* genes into the *YPRCΔ*15 locus to generate strain YH12. As shown in Figure 2B, the titer of naringenin was increased to 80.45 mg/L, 320% of strain YH1.

In order to reduce the formation of by-products BNY and CTAL, we chose four *CHILs* from different plants, namely, *SbCHIL*, *PhCHIL*, *AtCHIL*, and *MdCHIL*, which were inserted into plasmid pRS426, and then transformed into strain YH12 to generate strains YH13 to YH16. As shown in Figure 2B, feeding CA in medium, overexpression of SbCHIL exhibited a significant increase in naringenin (99.21 mg/L) and reduction of CTAL (Figure S3) compared with the absence of CHIL. Simultaneously, BNY formation was unchanged in most cases (Figure S3). As a result, *SbCHIL* combined with *Ha*4*CL*, *EbCHS*, and *ErCHI* were integrated into the *YPRCΔ*15 locus to generate strain YH18, which produced 110.32 mg/L of naringenin after 72 h fermentation, 140% of strain YH12.

### 3.2. Investigation of the Subcellular Localization of the Midstream Module

**Figure 3:**
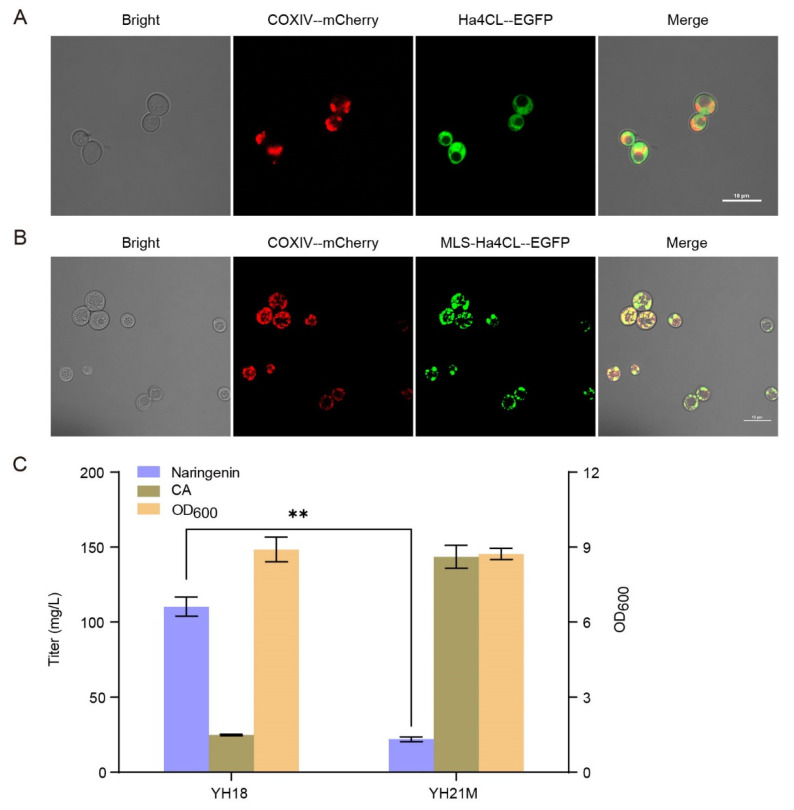
Localization of the midstream module in the mitochondria for the biosynthesis of naringenin from CA. (**A**) Fluorescence microscopy of strain YH20C. (**B**) Fluorescence microscopy of strain YH20M. “--” refers to the (GGGGS)_3_ linker. The results are imaged at a 60× magnification. Scale bars represent 10 μm. (**C**) Effect of the midstream module localization in the mitochondria on naringenin biosynthesis from CA (supplemented 200 mg/L). The data are the means and standard deviations of three replicates (**, *p* < 0.01; Student’s *t*-test).

As the most widely localized organelle in metabolic engineering, the mitochondria matrix of yeast has higher redox potential and more ATP than the cytosol. Thus, we tried to investigate the capacity of yeast mitochondria for naringenin production. Subunit IV of the yeast cytochrome oxidase (COXIV) is the native protein of mitochondria of *S. cerevisiae*. We first fused mCherry with COXIV to label mitochondria as red, obtaining strain YH19M. Then, using fused Ha4CL-EGFP as the target protein, we tested the guidance effect of the 26-amino acid N-terminal mitochondrial localization signal (MLS, LSLRQSIRFFKPATRTLCSSRYLLQ) from COXIV on the localization into the mitochondrial matrix. As shown in Figure 3A, under a confocal laser scanning microscope, the red fluorescence was distributed mostly in the mitochondria, and green fluorescence was distributed diffusely in the cytosol, indicating that Ha4CL was expressed in the cytosol of strain YH20C. The almost complete colocalization of COXIV-mCherry and MLS-Ha4CL-EGFP was observed on the inner membrane of mitochondria in strain YH20M, indicating that MLS-Ha4CL was successfully localized in the mitochondria (Figure 3B). Next, four genes (Ha4CL, EbCHS, ErCHI, SbCHIL) of the midstream module were localized in the mitochondria, generating strain YH21M. However, compared with strain YH18, the naringenin production of strain YH21M decreased sharply to 22.04 mg/L with a large amount of CA remaining (Figure 3C). Thus, the mitochondria is not suitable for flavonoid biosynthesis.

**Figure 4:**
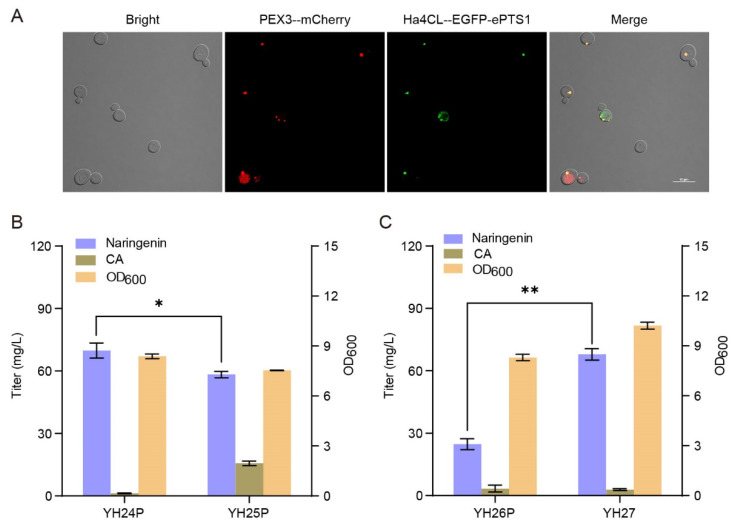
Localization of the midstream module in the peroxisomes for the biosynthesis of naringenin from CA. (**A**) Fluorescence microscopy of strain YH23P. “--” refers to the (GGGGS)_3_ linker. The results are imaged at a 60× magnification. Scale bars represent 10 μm. (**B**) Effect of the midstream module localization in the peroxisomes on naringenin biosynthesis from CA (supplemented 200 mg/L). (**C**) Comparison of naringenin production from tyrosine (supplemented 500 mg/L) between strains YH26P and YH27. The data are the means and standard deviations of three replicates (*, *p* < 0.05; **, *p* < 0.01; Student’s *t*-test).

The peroxisome is another potential organelle for heterologous biosynthesis of natural products by supplying more acetyl-CoA and NADPH. Analogously, the peroxisomal membrane protein PEX3-mCherry labeled the peroxisome as red. Then, using the enhanced C-terminal peroxisome targeting signal type 1 (ePTS1, LGRGRRSKL) to guide and localize the enzymes of the midstream module into the peroxisomes, we constructed strain YH23P expressing Ha4CL-EGFP-ePTS1 and strain YH24P expressing Ha4CL-ePTS1-EbCHS-ePTS1-ErCHI-ePTS1-SbCHIL-ePTS1. As shown in Figure 4A, Ha4CL-ePTS1 was successfully localized in the peroxisomes as the almost complete colocalization of PEX3-mCherry and Ha4CL-EGFP-ePTS1 was observed on the peripheral peroxisomes in strain YH23P. When CA was supplemented in medium, the naringenin titer of strain YH24P was 69.82 mg/L, 217% higher than strain YH21M (Figure 4B). Meanwhile, almost no CA was detected in the fermentation broth. To further improve the production of naringenin from CA in the peroxisomes, we tried to enhance the carbon flux from acetyl-CoA to malonyl-CoA by overexpressing the mutation of acetyl-CoA carboxylase (ACC1^AAA^), generating strain YH25P. Unfortunately, strain YH25P produced 58.30 mg/L of naringenin, 16% lower than strain YH24P (Figure 4B). It indicated that the overexpression of ACC1^AAA^-ePTS1 in the peroxisomes did not benefit the production of naringenin.

To further explore the feasibility of naringenin biosynthesis from tyrosine in the peroxisomes, we constructed strain YH26P by integrating the co-construct of *FjTAL-ePTS*1 and *EbCHS-ePTS*1 into the 1622*b* locus of strain YH24P. As shown in Figure 4C, strain YH26P produced 24.72 mg/L of naringenin from tyrosine. When the *FjTAL* and *EbCHS* genes were integrated into the 1622*b* locus of strain YH18, the resulting strain YH27 produced 67.90 mg/L of naringenin from tyrosine, 175% higher than strain YH26P. Taken together, although the production of naringenin in the peroxisomes is better than in the mitochondria, it is still less efficient than in the cytosol.

### 3.3. Efficient De Novo Biosynthesis of Naringenin

**Figure 5:**
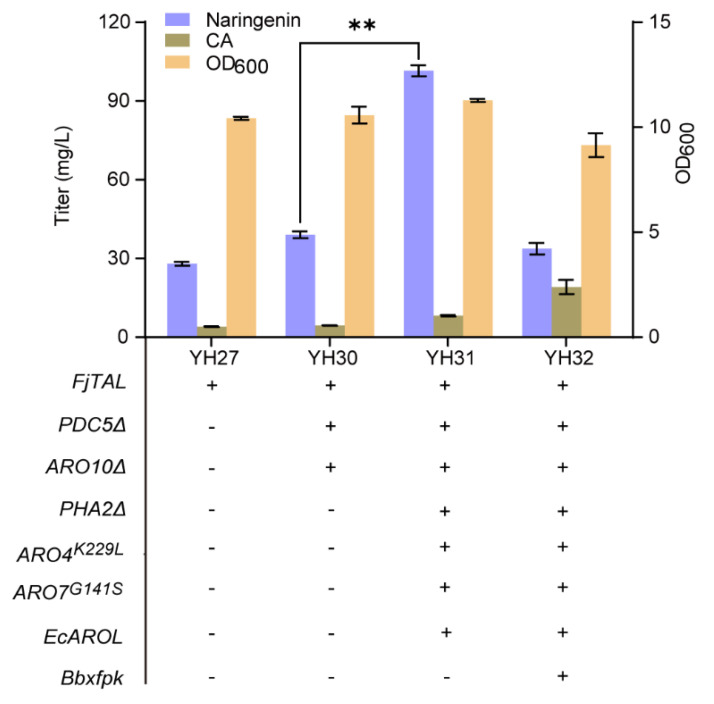
De novo biosynthesis of naringenin by rewiring metabolic pathway of tyrosine. The data are the means and standard deviations of three replicates (**, *p* < 0.01; Student’s *t*-test).

In order to increase the biosynthesis of naringenin from glucose, we rewired the synthetic pathway of precursor tyrosine in the upstream module (Figure 1). We first blocked the competing pathway by knocking out the *PDC*5 and *ARO*10 genes, and the resulting strain YH30 produced 39.05 mg/L of naringenin, a 40% increase compared with strain YH27 (28.01 mg/L). Secondly, to alleviate the feedback inhibition of DAHP synthase (ARO4) and chorismite mutase (ARO7) and to block the competing phenylalanine synthesis for enhancement of the metabolic flux toward tyrosine, we integrated the feedback-resistant *ARO*4*^K^*^229*L*^ and *ARO*7*^G^*^141*S*^, and *EcAROL* into the *PHA*2 locus of strain YH30, generating strain YH31. As shown in Figure 5, the titer of naringenin from glucose was improved to 101.58 mg/L in strain YH31, 260% of strain YH30. Thirdly, to enhance the flux of E4P from F6P which catalyzes by phosphoketolase (XFPK), we integrated the *Bbxfpk* gene from *Bifidobacterium breve* into the 308*a* locus of strain YH31 to obtain strain YH32. Unexpectedly, the titer of naringenin was significantly decreased to 33.73 mg/L in strain YH32, along with the inhibition of cell growth (Figure 5). Therefore, we used strain YH31 to produce genistein in the subsequent experiments.

### 3.4. De Novo Biosynthesis of Genistein

**Figure 6:**
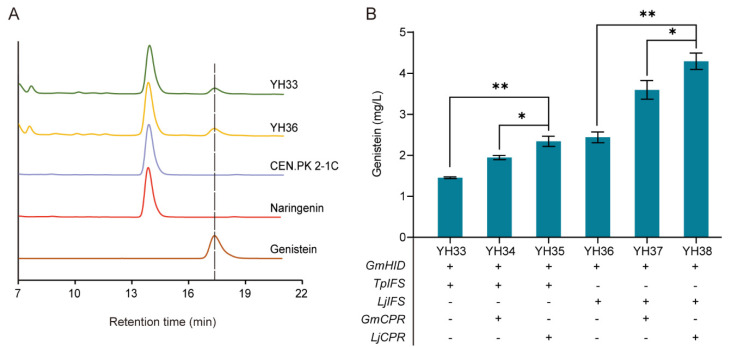
Biosynthesis of genistein from naringenin. (**A**) HPLC profile of strains YH33, YH36, and CEN.PK2-1C. (**B**) Combinatorial optimization of IFS-CPR pairs to improve genistein production at 72 h. 100 mg/L naringenin was supplemented at 0 h. The data are the means and standard deviations of three replicates (*, *p* < 0.05; **, *p* < 0.01; Student’s *t*-test).

In the downstream module, IFS and CPR are key enzymes for genistein biosynthesis from naringenin. To this end, two IFSs from *Trifolium pratense* (*TpIFS*) and *Lotus japonicas* (*LjIFS*) were coexpressed with the *GmHID* gene from *G. max* in the *YORW∆*17 locus of CEN. PK2-1C to generate strains YH33 and YH36, respectively. As shown in Figure 6A, without the heterologous CPR expression, strains YH33 and YH36 successfully produced genistein from naringenin. To improve the genistein production, two CPRs from *G. max* (*GmCPR*) and *L. japonicas* (*LjCPR*) were incorporated, generating strains YH34, YH35, YH37, and YH38. As shown in Figure 6B, the heterologous expression of CPR significantly improved the genistein production with whichever IFSs were used, indicating they favored the electron transfer to IFS. Despite LjIFS or TpIFS, LjCPR was more beneficial than GmCPR for the production of genistein, and among four IFS-CPR pairs, the expression of the LjIFS-LjCPR pair in strain YH38 led to the highest genistein titer of 4.30 mg/L.

**Figure 7:**
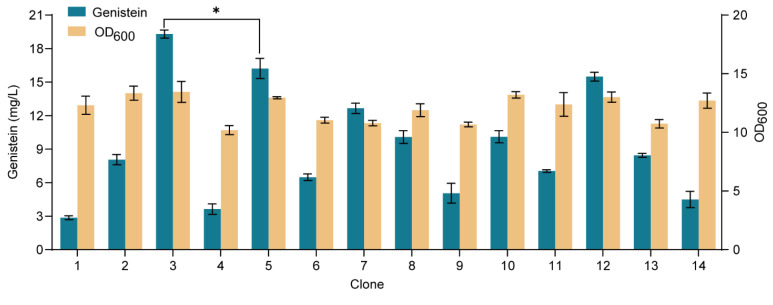
De novo biosynthesis of genistein in *S. cerevisiae*. Genistein titer and cell biomass were tested on the 14 clones at 72 h. The data are the means and standard deviations of three replicates (*, *p* < 0.05; Student’s *t*-test).

To achieve de novo biosynthesis of genistein, we integrated the *LjIFS*, *LjCPR*, and *GmHID* genes into the *YORW∆*17 locus of strain YH31, and the resulting strain YH39 produced 2.03 mg/L of genistein. It indicated that the low gene dosage of the downstream module might restrict the genistein biosynthesis. Next, we additionally integrated the *LjIFS* gene into the multicopy *delta* locus of strain YH39, and 14 positive clones were randomly screened (Figure 7). Among them, clone 3 produced 19.32 mg/L of genistein from glucose, 952% of strain YH39, and it was designated strain YH40, which was used for the following study.

### 3.5. Fermentation Optimization to Increase Genistein Production

**Figure 8:**
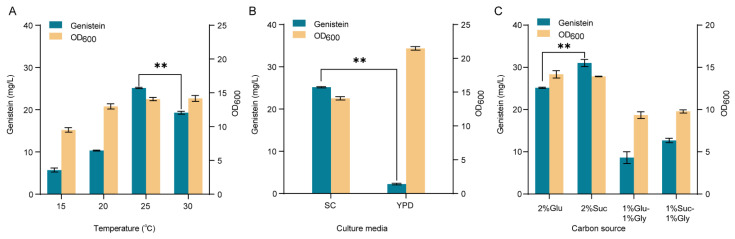
Fermentation optimization of the engineered strain YH40. (**A**) Effects of culture temperature on genistein production and cell biomass in SC medium. (**B**) Effects of culture media on genistein production and cell biomass at 25 °C. (**C**) Effects of different carbon sources on genistein production and cell biomass in SC medium at 25 °C. Glu, glucose; Suc, sucrose; Gly, glycerol. The data are the means and standard deviations of three replicates (**, *p* < 0.01; Student’s *t*-test).

To improve the genistein production, we optimized the culture temperature and fermentation medium. As shown in Figure 8A, the cultivation of strain YH40 at 25 °C showed the highest genistein titer of 25.16 mg/L, 130% of that at 30 °C, and there was no significant difference in biomass. However, the titer of genistein and cell biomass was significantly reduced when strain YH40 was cultivated at 20 °C and 15 °C. Then, we tried to replace the SC medium with an inexpensive YPD medium for the production of genistein at 25 °C. As shown in Figure 8B, the growth of strain YH40 was greatly improved in the YPD medium, while the titer of genistein was sharply decreased to 2.25 mg/L, indicating the imbalance between cell growth and genistein biosynthesis.

Furthermore, in addition to glucose, sucrose is cheaper, and glycerol provides a higher degree of reduction, which is needed for the enzymatic reaction of IFS and CPR. To verify their effects on genistein production in *S. cerevisiae*, we used the sole glucose, sole sucrose, glucose and glycerol mixture, and sucrose and glycerol mixture as carbon sources. As shown in Figure 8C, using sucrose as the sole carbon source improved the titer of genistein to 31.02 mg/L, a 20% increase compared with using sole glucose. While incorporating glycerol into glucose or sucrose as mixed carbon sources did not improve genistein production and cell biomass of strain YH40.

## 4. Discussion

As an important flavonoid scaffold, naringenin is converted from CA catalyzed by three enzymes 4CL, CHS, and CHI. Although the production of naringenin has been extensively studied in yeast, the efficiency remains unsatisfactory [22,23], and the major bottleneck is the poor activity and promiscuity of CHS. In the midstream module, we first screened out the most efficient EbCHS to improve the production of naringenin, indicating that EbCHS might possess higher activity than other CHSs tested in yeast. Then, we additionally employed CHIL, an auxiliary protein with a non-catalytic function, which can rectify the derailment of the CHS-catalyzed reaction and guide metabolic flux into naringenin [9,10]. The optimal SbCHIL interacted with EbCHS, effectively increasing the titer of naringenin and simultaneously reducing the formation of the by-product CTAL in engineered *S. cerevisiae*. Recently, we showed that CHIL is also efficient for naringenin production in engineered *E. coli* [18]. CHIL might be a necessary component for the higher production of naringenin in the flavonoid biosynthetic pathway.

Organelle engineering, an emerging strategy in synthetic biology, and metabolic engineering [24] have been employed in yeast to produce natural products, such as hydrocortisone [25], squalene [26,27], branched-chain alcohols [28], and alkaloids [29]. However, few attempts have been focused on the organelle engineering of flavonoid compounds. We localized the midstream module in the mitochondria and peroxisomes to explore the possibility of naringenin production. Our results and previous work [30] showed that the mitochondria are not suitable for flavonoid biosynthesis. One possible explanation was that CA could not enter the mitochondrial matrix due to poor permeability. Although naringenin production in the peroxisomes was more favorable than in the mitochondria, it was still less efficient than in the cytosol, the possible reason may be that the microenvironment of peroxisomes is not conducive to the functionality of naringenin pathway enzymes, and the underlying mechanisms need to be further explored in further studies.

Increasing the availability of the precursor tyrosine is another strategy to enhance naringenin production. In our work, the titer of naringenin was significantly increased by knocking out PDC5, ARO10, and PHA2 and overexpressing ARO4K229L, ARO7G141S, and EcAROL in the upstream module. However, when Bbxfpk was incorporated to divert part of the carbon flux from glycolysis to E4P formation, an adverse effect was observed on naringenin production, which might be because the supply of E4P is currently sufficient for the synthesis of naringenin.

In the downstream module, IFS belongs to the P450 family and may not be fully activated by yeast endogenous CPR. In our work, two IFSs and two CPRs were tested, and the optimal combination of LjIFS and LjCPR remarkably increased genistein production, which was consistent with previous reports that the homologous CYP-CPR combination was more efficient than the heterologous one in yeast [31,32]. Moreover, high expression of the downstream module in multiple copies of the *delta* locus on the chromosomes was feasible to increase the titer of genistein. The suitable temperature for genistein production was lower than that for yeast growth, which was consistent with the previous report that the suboptimal growth temperature benefited the production of genistein from naringenin in *E. coli* [33]. Previous works showed that the mixture of glucose and glycerol increased the production of genistein in *E. coli* [18] and that the mixture of sucrose and glycerol increased the production of kaempferol in yeast [30], while our work showed sucrose as the sole carbon was better than sole glucose, followed by a mixture of sucrose and glycerol, or a mixture of glucose and glycerol to the biosynthesis of genistein in yeast. It might be that different carbon sources have various physiological effects on flavonoid biosynthesis in different microbes, which need to be explored further.

In conclusion, we for the first time reported the de novo biosynthesis of genistein by modular engineering of *S. cerevisiae*. The novel midstream module was composed of Ha4CL, EbCHS, ErCHI, and SbCHIL and greatly contributed to the production of naringenin and the reduction of by-product formation. Knocking out the competing phenylalanine and *p*-hydroxyacetaldehyde biosynthetic branch and removing the feedback inhibition of the tyrosine synthetic pathway improved the carbon metabolic flux into CA in the upstream module. Furthermore, the homologous LjIFS-LjCPR pair in the downstream module was better than non-homologous pairs for the production of genistein from naringenin. Under optimized fermentation conditions, the final engineered strain YH40 integrated three modules produced 31.02 mg/L of genistein from sucrose at 25 °C. Our results provide valuable insights into the heterologous production of flavonoids and isoflavonoids in industrial microbes.

## Figures and Tables

**Table 1:** Strains used in this study.

Strains	Description	Source
CEN.PK2-1C	*MATa*; *ura*3*-*52, *trp*1*-*289, *leu*2*-*3112, *his*3*Δ*1, *MAL*2*-*8*C*, *SUC*2	Invitrogen
YH1	CEN.PK2-1C, pMNG1	This study
YH2	CEN.PK2-1C, pMNG2	This study
YH3	CEN.PK2-1C, pMNG3	This study
YH4	CEN.PK2-1C, pMNG4	This study
YH5	CEN.PK2-1C, pMNG5	This study
YH6	CEN.PK2-1C, pMNG6	This study
YH7	CEN.PK2-1C, pMNG7	This study
YH8	CEN.PK2-1C, pMNG8	This study
YH12	CEN.PK2-1C, *YPRC∆*15*::loxp*-*LEU*2-*loxp*-P*_HXT_*_7_-*EbCH*S-T*_ADH_*_1_-P*_TEF_*_1_-*Ha*4*CL*-T*_GPM_*_1_-P*_TEF_*_2_-*ErCHI*-T*_CYC_*_1_	This study
YH13	YH12, pMNG12	This study
YH14	YH12, pMNG13	This study
YH15	YH12, pMNG14	This study
YH16	YH12, pMNG15	This study
YH18	CEN.PK2-1C, *YPRC∆*15*::loxp*-*LEU*2-*loxp*-P*_HXT_*_7_-*EbCH*S-T*_ADH_*_1_-P*_TEF_*_1_-*Ha*4*CL*-T*_GPM_*_1_-P*_TEF_*_2_-*ErCHI*-T*_CYC_*_1_*-*P*_PGK_*_1_*-SbCHIL-*T*_GPD_*	This study
YH19M	CEN.PK2-1C, *HO::*HIS3-P*_TDH_*_3_*-COXIV*-(GGGGS)_3_-*mCherry*-T*_ACS_*_1_	This study
YH20C	YH19M, pMNG17	This study
YH20M	YH19M, pMNG18	This study
YH21M	CEN.PK2-1C, *YPRC∆*15*::loxp*-*LEU*2-*loxp*-P*_HXT_*_7_-*MLS*-*EbCH*S-T*_ADH_*_1_-P*_TEF_*_1_-*MLS*-*Ha*4*CL*-T*_GPM_*_1_-P*_TEF_*_2_-*MLS*-*ErCHI*-T*_CYC_*_1_*-*P*_PGK_*_1_-*MLS-SbCHIL-*T*_GPD_*	This study
YH22P	CEN.PK2-1C, *HO::*HIS3-P*_TDH_*_3_*-PEX*3-(GGGGS)_3_-*mCherry*-T*_ACS_*_1_	This study
YH23P	YH22P, pMNG19	This study
YH24P	CEN.PK2-1C, *YPRC∆*15*::loxp*-*LEU*2-*loxp*-P*_HXT_*_7_-*EbCH*S-*ePTS*1-T*_ADH_*_1_-P*_TEF_*_1_-*Ha*4*CL-ePTS*1-T*_GPM_*_1_-P*_TEF_*_2_-*ErCHI-ePTS*1-T*_CYC_*_1_*-*P*_PGK_*_1_-*SbCHIL-ePTS*1*-*T*_GPD_*	This study
YH25P	YH24P, *HO::loxp-TRP*1*-loxp*-P*_TDH_*_1_-*ACC*1*^S^*^659*A*,*S*686*A*,*S*1157*A*^-*ePTS*1-T*_ACS_*_1_	This study
YH26P	YH24P, 1622*b::loxp-HIS*3*-loxp*-P*_HXT_*_7_-*EbCHS-ePTS*1-T*_ADH_*_1_-P*_TDH_*_3_-*FjTAL-ePTS*1-T*_ENO_*_2_	This study
YH27	YH18, 1622*b::loxp-HIS*3*-loxp*-P*_HXT_*_7_-*EbCHS*-T*_ADH_*_1_-P*_TDH_*_3_-*FjTAL*-T*_ENO_*_2_	This study
YH28	YH27, *PDC*5Δ::*loxp-TRP*1*-loxp*	This study
YH29	YH28, pCRE	This study
YH30	YH29, *ARO*10Δ::*loxp-HIS*3*-loxp*	This study
YH31	YH30, *PHA*2Δ*::loxp-LEU*2*-loxp*-P*_TDH_*_3_-*ARO*4*^K^*^229*L*^-T*_ADH_*_1_-P*_HXT_*_7_-*ARO*7*^G^*^141*S*^-T*_TEF_*_1_-P*_HXK_*_1_-*EcAROL*-T*_CYC_*_1_	This study
YH32	YH31, 308*a::loxp-TRP*1*-loxp*-P*_TDH_*_1_-*Bbxfpk*-T*_GPD_*	This study
YH33	CEN.PK2-1C, *YORW∆*17*::loxp-TRP*1*-loxp*-P*_TDH_*_1_-*TpIFS*-T*_PGK_*_1_-P*_CCW_*_12_-*GmHID*-T*_TEF_*_1_	This study
YH34	CEN.PK2-1C, *YORW∆*17*::loxp-TRP*1*-loxp*-P*_TDH_*_1_-*TpIFS*-T*_PGK_*_1_-P*_PDC_*_1_-*GmCPR*-T*_TDH_*_2_-P*_CCW_*_12_-*GmHID*-T*_TEF_*_1_	This study
YH35	CEN.PK2-1C, *YORW∆*17*::loxp-TRP*1*-loxp*-P*_TDH_*_1_-*TpIFS*-T*_PGK_*_1_-P*_PDC_*_1_-*LjCPR*-T*_TDH_*_2_-P*_CCW_*_12_-*GmHID*-T*_TEF_*_1_	This study
YH36	CEN.PK2-1C, *YORW∆*17*::loxp-TRP*1*-loxp*-P*_TDH_*_1_-*LjIFS*-T*_PGK_*_1_-P*_CCW_*_12_-*GmHID*-T*_TEF_*_1_	This study
YH37	CEN.PK2-1C, *YORW∆*17*::loxp-TRP*1*-loxp*-P*_TDH_*_1_-*LjIFS*-T*_PGK_*_1_-P*_PDC_*_1_-*GmCPR*-T*_TDH_*_2_-P*_CCW_*_12_-*GmHID*-T*_TEF_*_1_	This study
YH38	CEN.PK2-1C, *YORW∆*17*::loxp-TRP*1*-loxp*-P*_TDH_*_1_-*LjIFS*-T*_PGK_*_1_-P*_PDC_*_1_-*LjCPR*-T*_TDH_*_2_-P*_CCW_*_12_-*GmHID*-T*_TEF_*_1_	This study
YH39	YH31, *YORW∆*17*::loxp-TRP*1*-loxp*-P*_TDH_*_1_-*LjIFS*-T*_PGK_*_1_-P*_PDC_*_1_-*LjCPR*-T*_TDH_*_2_-P*_CCW_*_12_-*GmHID*-T*_TEF_*_1_	This study
YH40	YH39, *delta*::*loxp-URA*3*-loxp*-P*_TDH_*_1_-*LjIFS*-T*_PGK_*_1_	This study

## Data Availability

All data are present in the manuscript and its Supplementary Materials.

## Supplementary Materials

The following supporting information can be downloaded at: https://www.mdpi.com/article/10.3390/microorganisms10071402/s1, Figure S1: The pedigree chart of yeast strains constructed in this study; Figure S2: The accumulation of CA by strains expressing CHSs from different plants in SC-Ura media after 72 h fermentation; Figure S3: The effects of CHILs on the formation of by-products BNY and CTAL; Table S1: The sources of heterologous genes used in this study; Table S2: Nucleotide sequences of codon-optimized genes; Table S3: The main primers used in this study; Table S4. Plasmids used in this study. Supplementary File S1: Figures S4–S9, The maps of key plasmids. Table S5, The sequences of promoters and terminators used in this study.
