# Supplementary material for: Modular Engineering of Saccharomyces cerevisiae for De Novo Biosynthesis of Genistein
Source: Microorganisms. 2022 Jul 12;10(7):1402. doi: 10.3390/microorganisms10071402 (PMC9319343; doi:10.3390/microorganisms10071402)
Supplement: Supplementary file 1 [file microorganisms-10-01402-s001.zip › microorganisms-1796123-supplementary.pdf]

Supplementary Materials

Modular Engineering of *Saccharomyces cerevisiae* for De Novo Biosynthesis of Genistein

Yonghui Meng [1,2], Xue Liu [1,2], Lijuan Zhang [1,2] and Guang-Rong Zhao [1,2,*]

[1] Frontiers Science Center for Synthetic Biology and Key Laboratory of Systems Bioengineering (Ministry of Education), School of Chemical Engineering and Technology, Tianjin University, Yaguan Road 135, Jinnan District, Tianjin 300350, China; mengyonghui_2019@tju.edu.cn (Y.M.); hiliuxue@tju.edu.cn (X.L.); zyljzhang@tju.edu.cn (L.Z.)

[2] Georgia Tech Shenzhen Institute, Tianjin University, Dashi Road 1, Nanshan District, Shenzhen 518055, China

[*] Correspondence: grzhao@tju.edu.cn; Tel.: +86-22-85356580; Fax: +86-22-27403389

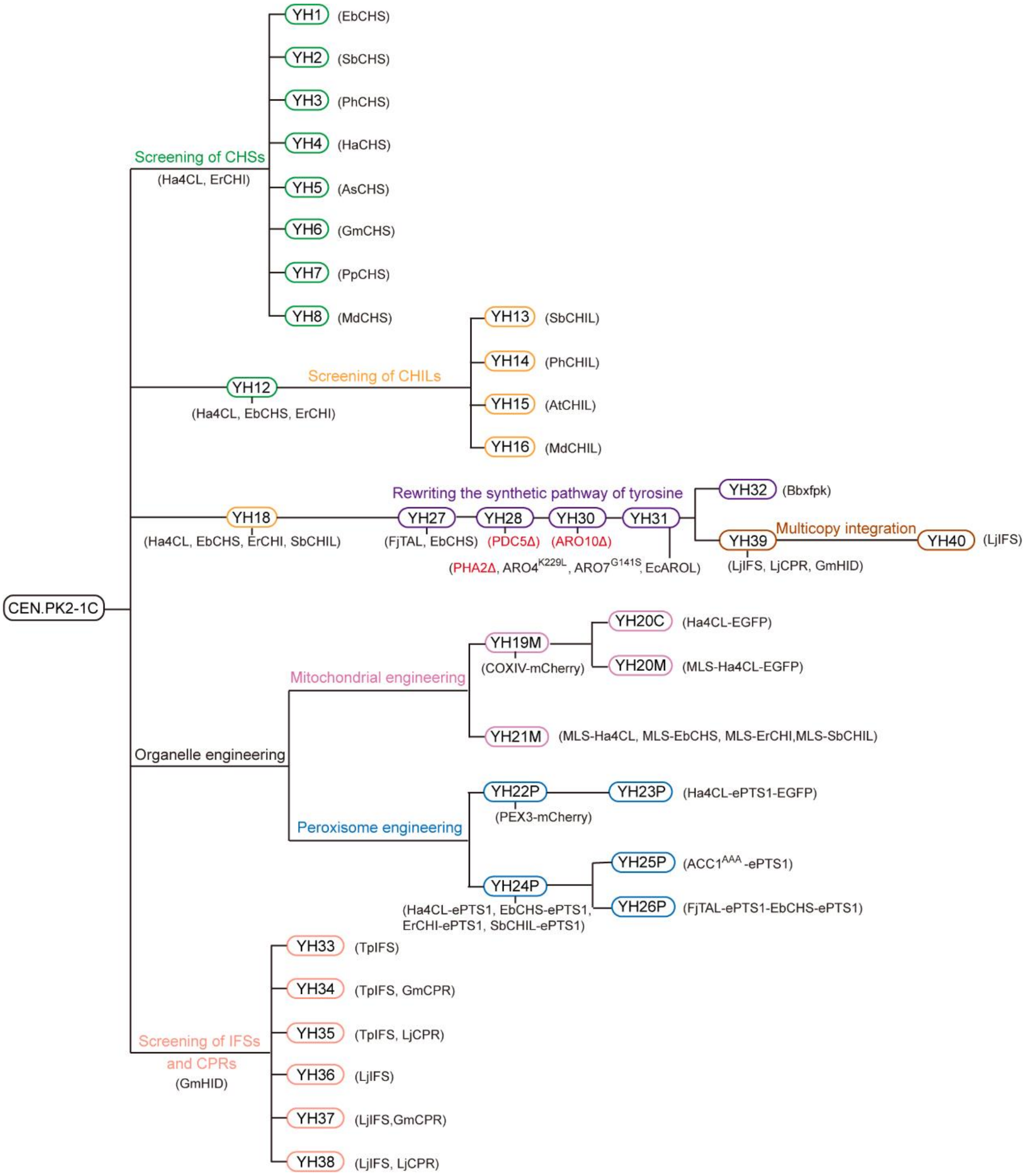

Figure S1. The pedigree chart of yeast strains constructed in this study.

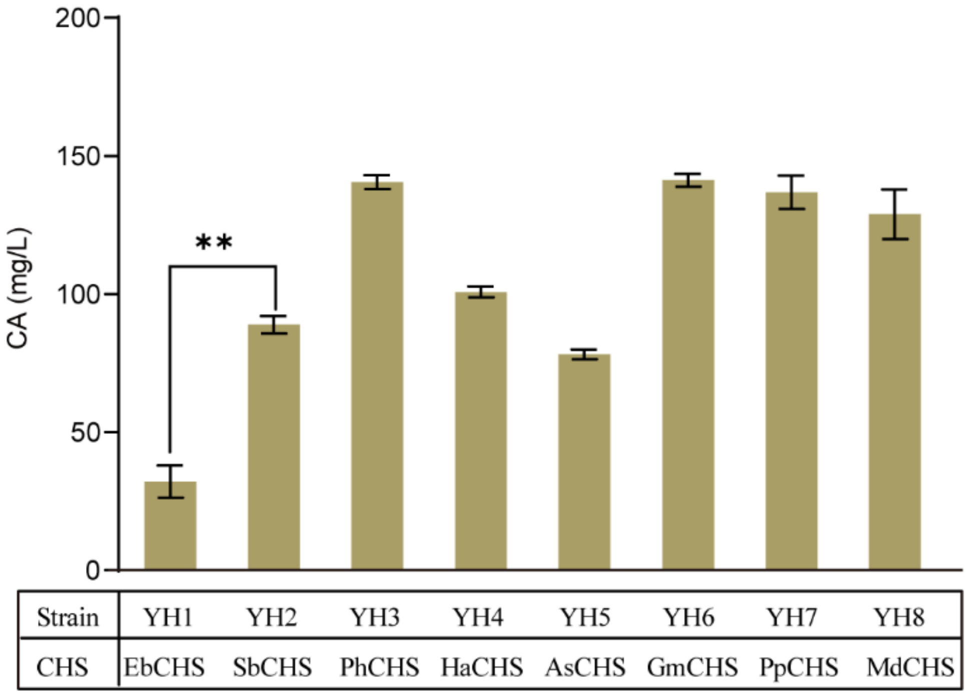

Figure S2. The accumulation of CA by strains expressing CHSs from different plants in SC-Ura media after 72 h fermentation. 200 mg/L CA was supplemented at 0 h. The data are the means and standard deviations of three replicates (**, $p < 0.01$; Student's t-test).

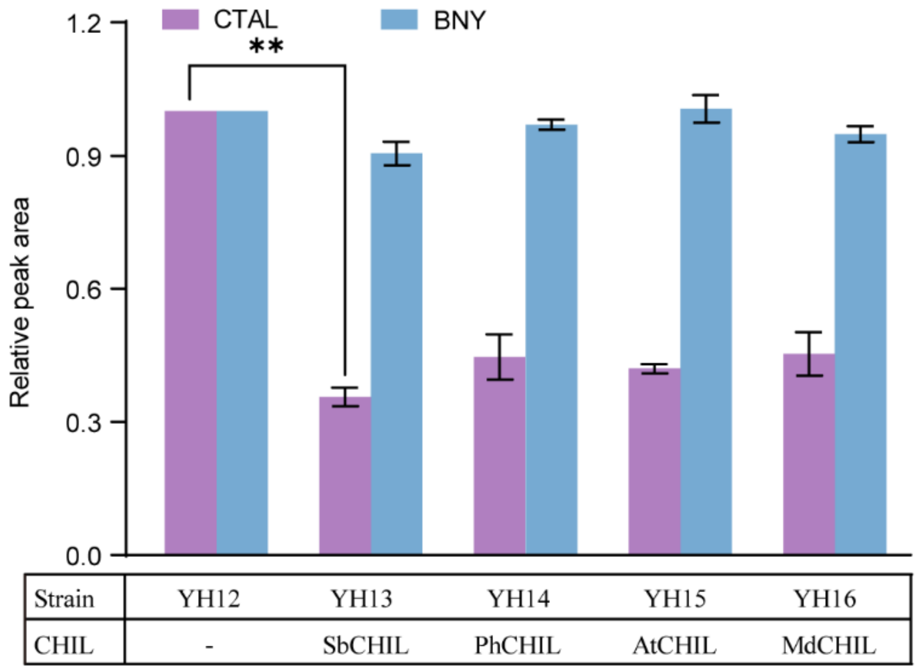

Figure S3. The effects of CHILs on the formation of by-products BNY and CTAL. The productions of BNY and CTAL normalized to that of strain YH12. The data are the means and standard deviations of three replicates (**, $p < 0.01$; Student's t-test).

Table S1. The sources of heterologous genes used in this study.

**Gene**	**Source**
FjTAL	*Flavobacterium johnsoniaeu*
Ha4CL	*Helianthus annuus*
EbCHS	*Erigeron breviscapus*
SbCHS	*Sorghum bicolor*
PhCHS	*Petunia x hybrida*
HaCHS	*Hypericum androsaemum*
AsCHS	*Aquilaria sinensis*
GmCHS	*Glycine max*
PpCHS	*Pyrus pyrifolia*
MdCHS	*Malus domestica*
SbCHIL	*Sorghum bicolor*
PhCHIL	*Petunia x hybrida*
AtCHIL	*Arabidopsis thaliana*
MdCHIL	*Malus domestica*
ErCHI	*Eubacterium ramulus*
Bbxfpk	*Bifidobacterium breve*
LjIFS	*Lotus japonicas*
TpIFS	*Trifolium pratense*
GmCPR	*Glycine max*
LjCPR	*Lotus japonicas*
GmHID	*Glycine max*

Table S2. Nucleotide sequences of codon-optimized genes.

Gene	Sequence (5′-3′)
FjTAL	ATGAACACCATCAACGAGTACCTGTCTCTGGAAGAATTTGAAGCTATCATCTTCGGTAACCAGAAGGTTACT<br>ATCTCCGATGTTGTTGTTAACAGAGTTAACGAATCTTTCAACTTCTTGAAGGAATTTTCTGGTAACAAGGTTAT<br>CTACGGTGTTAACACTGGTTTCGGTCCAATGGCTCAATATAGAATCAAGGAGTCTGATCAAATCCAATTGCA<br>ATACAACTTGATCAGATCACATTCTTCTGGTACAGGTAAACCATTGTCCCCAGTTTGTGCTAAAGCTGCTATTT<br>TGGCTAGGCTGAACACCTTGTCCCTGGGTAATTCTGGTGTCCATCCATCTGTCATCAACCTGATGTCCGAGTT<br>GATCAACAAGGACATCACCCCACTGATCTTCGAGCATGGTGGTGTTGGTGCTTCTGGTGATTTGGTTCAGCTG<br>TCTCACCTGGCTTTGGTTTTGATCGGTGAGGGTGAAGTCTTCTACAAGGGTGAAAGGAGGCCAACACCAGA<br>AGTTTTCGAGATCGAGGGTCTGAAGCCAATCCAAGTTGAGATCAGGGAGGGTTTAGCTTTGATCAACGGTAC<br>ATCCGTCATGACAGGTATCGGTGTTGTTAACGTCTACCACGCTAAGAAGCTGCTGGATTGGTCTTTGAAGTCC<br>TCCTGTGCTATCAACGAATTGGTTCAAGCATACGATGATCATTTCTCTGCTGAATTGAACCAGACAAAGAGG<br>CACAAGGGCCAACAAGAAATCGCTTTGAAGATGAGGCAGAACCTGTCCGATTCCACTTTGATCAGGAAGAG<br>GGAGGACCATCTGTACTCTGGTGAAAACACCGAGGAGATTTTTAAGGAGAAGGTCCAGGAGTACTACTCCT<br>TGAGATGCGTTCCACAGATCCTGGGTCCAGTTTTGGAAACTATCAACAACGTCGCTTCCATCCTGGAGGATG<br>AATTTAACTCCGCCAACGACAACCCCATCATCGATGTTAAGAACCAGCACGTCTACCACGGTGGTAATTTCC<br>ACGGTGATTACATCTCTTTGGAAATGGATAAGTTGAAGATCGTTATCACTAAGTTGACTATGCTGGCTGAAAG<br>ACAATTGAACTACTTGTTGAACTCTAAGATCAACGAGTTACTGCCCCCATTCGTCAACTTGGGTACTTTAGGT<br>TTCAACTTCGGTATGCAGGGTGTCCAATTCACCGCTACATCTACAACCGCTGAGTCTCAAATGCTGTCCAACC<br>CAATGTACGTCCACTCTATCCCAAACAACAACGACAACCAGGACATCGTCTCTATGGGTACTAACTCCGCTG<br>TCATCACCTCTAAGGTCATCGAAAACGCCTTCGAGGTCTTGGCTATCGAAATGATCACCATCGTCCAGGCTAT<br>CGACTACTTGGGTCAAAAGGACAAGATCAGCTCCGTCTCTAAGAAGTGGTACGACGAAATCAGGAACATCA<br>TCCCCACCTTCAAGGAGGATCAGGTTATGTACCCCTTCGTCCAAAAGGTCAAGGACCATTTGATCAACAACT<br>AA
Ha4CL	ATGGCTCCAGAAAAGGAAATCATCTTCAGATCCAAGTTGCCAGACATTTACATCCCAAAGCACCTCCCCCTA<br>CATTCTTACTGTTTCGAAAACATCTCTACTTTCAACAACAGACCATGCTTGATCGATGGTGCTACCGGTGTCG<br>TCCACACTTACGCTGACGTTGAACTAACATCTCGTAAGGTTGCCTCTGCTTTCCACCAACACGGTATCAACA<br>AGGGTGATGTTATCATGATTTTATTGCCAAACTCTCCAGAATTTGTTTACTCTTTCTTCGGTGCCTCCTATTTGG<br>GTGCCGTATCAACTATGGCTAACCCTTTTTTCACTTCCGCTGAAATCATTAAACAAGCTAAGGCTTCCAATGC<br>TAAGATCATTGTCACTCAAGCCTCTCATGTTCCAAAGATCAAGGAATACGCTGCCGAAAATTCCATTAAGAT<br>CGTCTGTATCGATTCTGCTCCAGAAGGTTGTTTGCACTTCACTGAATTGGTCTCCGGTGACGAAACCAAGTTG<br>CCGGAAGTCGAAATTTCCTCCGACGATGTTGTTGCTTTGCCATACTCCTCTGGTACCACTGGGTTGCCAAAGG<br>GTGTTATGTTGACTCACAAAGGCTTGGTCACTTCTGTCGCTCAACAAGTTGACGGTGAAAACCCAAACTTGT<br>GGATTCACTCTGAAGACGTTCTGATGTGTGTTCTGCCATTGTTCCATATCTACTCTTTGAACAGCATCTTGTTG<br>TGTGGTTTGCGTGCTGGTGCTGCTATTTTGATTATGCAAAAGTTCGATATTGTGCCTTTCTTGGAATTGATTGA<br>AAAGTATAAGGTTACCATCGGTCCATTCGTTCCACCAATTGTTTTGGCCATTGCTAACAATGAAGAAGTTGTT<br>GACAAGTACGATTTGTCTTCCATGAGAACCGTCATGTCTGGTGCTGCTCCATTAGGTAAAGAATTGGAAGAC<br>ATGGTTAGAAGAAAGTTTACCAACGCTAAGCTAGGTCAAGGTTACGGTATGACTGAAGCCGGTCCAGTTTTG<br>GCTATGTGTTTGGCTTTCGCCAAGGAACCATACGAAATCAAGTCTGGTGCTTGTGGTACTGTCGTCAGAAAC<br>GCCGAAATGAAAATCGTTGATCCAGAAACTGGTTTGAGTTTACCAAGAAACCAAAGAGGTGAAATCTGTAT<br>CAGAGGTGACCAAATCATGAAGGGTTACTTGAACGACCCAGAATCTACCAAGACCACAATTGATTCCGATG<br>GTTGGTTGCACACCGGTGACATTGGTTTGATTGATGACGACGATGAATTATTTATTGTCGACAGATTGAAGGA<br>GTTAATCAAGTACAAGGGTTTCCAAGTTGCTCCAGCTGAATTGGAAGCCTTGTTGTTGACCCACCCACAAAT

TTCTGACGTTGCTGTCGTTCCAATGGTTAACGAAGCTGCTGGAGAAGTCCCAGTCGCCTTTGTTGTCAAGACT
AAGGACTCTAGTGTTACTGAAGATGACATCAAGCAATTCGTTCACAAGCAAGTCGTCTTCTACAAGAGAATC
AACAGAGTTTTCTTCATCGACACTATTCCAAAATCCCCTGCTGGTAAGATTTTGAGAAAGGAATTACGTGCTA
AATTAGCCGCCGGTGTCCCAAACTGA

EbCHS ATGGCTTCTTCTATCGACATCGCTGCTATCAGAGAAGCTCAAAGAGCTCAAGGTCCAGCTACTATCTTGGCTA
TCGGTACTGCTACTCCATCTAACTGTGTTTACCAAGCTGACTACCCAGACTACTACTTCAGAATCACTAAGTC
TGAACACATGGTTGACTTGAAGGAAAAGTTCAAGAGAATGTGTGACAAGTCTATGATCAGAAAGAGATACA
TGCACTTGACTGAAGAATACTTGAAGGAAAACCCATCTTTGTGTGAATACATGGCTCCATCTTTGGACGCTA
GACAAGACGTTGTTGTTGTTGAAGTTCCAAAGTTGGGTAAGGAAGCTGCTACTAAGGCTATCAAGGAATGG
GGTCAACCAAAGTCTAAGATCACTCACTTGATCTTCTGTACTACTTCTGGTGTTGACATGCCAGGTGCTGACT
ACCAATTGACTAAGTTGTTGGGTTTGAGACCATCTGTTAAGAGATTCATGATGTACCAACAAGGTTGTTTCGC
TGGTGGTACTGTTTTGAGATTGGCTAAGGACTTGGCTGAAAACAACAAGGGTGCTAGAGTTTTGGTTGTTTGT
TCTGAAATCACTGCTGTTACTTTCAGAGGTCCAAACGACACTCACTTGGACTCTTTGGTTGGTCAAGCTTTGT
TCGGTGACGGTGCTGCTGCTGTTATCGTTGGTTCTGACCCAGACTTGACTACTGAAAGACCATTGTTCGAAAT
GATCTCTGCTGCTCAAACTATCTTGCCAGACTCTGAAGGTGCTATCGACGGTCACTTGAGAGAAGTTGGTTTG
ACTTTCCACTTGTTGAAGGACGTTCCAGGTTTGATCTCTAAGAACATCGAAAAGGCTTTGACTCAAGCTTTCT
CTCCATTGGGTATCTCTGACTGGAACTCTTTGTTCTGGATCGCTCACCCAGGTGGTCCAGCTATCTTGGACCA
AGTTGAATTGAAGTTGGGTTTGAAGGAAGAAAAGATGAGAGCTACTAGACACGTTTTGTCTGAATACGGTA
ACATGTCTTCTGCTTGTGTTTTGTTCATCATCGACGAAATGAGAAAGAAGTCTGCTGAAGACGGTGCTGCTAC
TACTGGTGAAGGTTTGGACTGGGGTGTTTTGTTCGGTTTCGGTCCAGGTTTGACTGTTGAAACTGTTGTTTTGC
ACTCTTTGCCAACTACTACTGCTATCGCTACTTAA

ErCHI ATGGCTGATTTCAAGTTCGAACCAATGAGATCCTTGATTTACGTTGATTGTGTTTCCGAAGATTACAGACCAA
AGTTGCAAAGATGGATTTACAAGGTTCATATTCCAGATTCCATTTCCCAATTCGAACCATACGTTACCAAGTA
CGCTTTCTACCCATCCTTCCCAATTCCACCACAAGGTGATAGATTCGGTTACGCTAGAATGCAATTGACCGAA
CATCATTGGTTGGTTTCCGATTTGGACCCTAGATTGGAAATTAAGGCTATTGCTGAAACCTTCCCAATGGATG
TTTTGGTTTGGCAAGGTCAAATTCCAGCTGCTGCTCATACCGATGCTCAAATTGATTCCGATGGTGATGCTGG
TAACGCTGCTAGAAAGTCCAACAACGCTGAAGGTAACCCATTCATTTTCGCTTTCTTGCCAATGTGGTGGGA
AAAGGATTTGAAGGGTAAGGGTAGAACCATTGAAGATGGTGCTAACTACAGATTCAACATGACCATTGGTTT
CCCAGAAGGTGTTGATAAGGCTGAAGGTGAAAAGTGGTTGTTCGAAAAGGTTGTTCCAATTTTGCAAGCTG
CTCCAGAATGTACCAGAGTTTTGGCTTCCGCTGTTAAGAAGGATATTAACGGTTGTGTTATGGATTGGGTTTTG
GAAATTTGGTTCGAAAACCAATCCGGTTGGTACAAGGTTATGGTTGATGATATGAAGGCTTTGGAAAAGCCA
TCCTGGGCTCAACAAGATGCTTTCCCATTCTTGAAGCCATACCATAACGTTTGTTCCGCTGCTGTTGCTGATTA
CACCCCATCCAACAACTTGGCTAACTACAGAGGTTACATTACCATGAGATAA

SbCHIL ATGTTGATCTCCGCTGTCGGCTCTGAAACCAAGACCATTACTTTCGAAGGTATCCCATTCCCAGCTGAAATCA
CAGCTGCTGGTAACCCATTGTCCTTGTTGGCTACTGGTATTACCGACATTGAAATTCACTTCTTGCAAATCAA
ATACAACGCTATTGGTGTTTATTTGCACTCTAACGATGACTCCGATTTATTAACTACTCACTTAGGTGCTTGGA
AGGGTAAGACTGCTGAAGATTTGTTGGCAGATGCTGCTTTCTGGTCTGCTTTGGTTTCATCCCCTGTCGAAAA
GTTGCTAAGAGTTGTCGTCATCAAGGAGATCAAGGGTTCTCAATACGGTGTTCAATTGGAATCTTCTGTTAGA
GACAGATTGGCCGCTGTTGACTTGTACGAAGACGATGAAGAAGAAGCTTTAGAAAAAGTTGCTGAATTTTTC
CAAGCCAAGTACTTCAAGCCAGGTTCCGTTATCACCTTTCATTTCCCAGCCACTCCAGGTCCAGCTGAAATTA
CCTTTGTCACTGAAGGTAAGGCTGATGCCAAGATCACTGTTGAAAACGAACACGTTGCCGGTATGATTCAAA
AGTGGTACTTGGGTGGTGACAATGCTGTCTCTCCAACCACCGTCCGTTCTTTGGCTGACAGATTCGCTGCTTT
GCTCGCCGCCTGA

PhCHIL ATGGGTAAGAACGAAGTTATGGTTGATGAAATTCCATTCCCATCCCAATTCATGATGACCACCAAGCCATTGC

CATTGATGGGTCATGGTATTACCGATATTGAAATTCATTTCTTGCAAATTAAGTTCACCGCTATTGGTGTTTACT
TGGACCCTGAAATTGTTACCCATTTGCAACAATGGAAGGGTAAGTCCGGTGCTGAATTGATTGAAAACGATG
AATTTTTCGAAGCTATTGTTAACGCTCCAGTTGATAAGTTCTTGAGAGTTGTTGTTATTAAGGAAATTAAGGGT
TCCCAATACGGTGTTCAATTGGAATCCGCTGTTAGAGATAGATTGGCTGAAGTTGATAAGTACGAAGAAGAA
GAAGAAGAAGCTCTTGAAAAGATTGTTGAATTTTTCCAATCCAAGTACTTCAAGAAGGATTCCGTTGTTACC
TACTCCTTCCCAGCTACCTCCGGTAACGTTAAGATTTCCTTCGCTACCGAAGGTAAGGAAGATTCCGAAATTG
AAGTTCAAAACGCTAACGTTGCTGGTGAAATTAAGAAGTGGTACTTGGGTGGTTCCAGAGGTTTGTCCCCAA
CCACCATTTCCTCCTTGGCTAACACCTTGTCCGCTGAATTGTCCAAGTAA

Bbxfpk ATGACTTCTCCAGTTATCGGTACTCCCTGGAAGAAGTTGAACGCTCCAGTTTCTGAAGAATCTTTGGAAGGT
GTTGACAAGTACTGGAGAGTTGCTAACTACTTGTCTATCGGTCAAATCTACTTGAGATCGAACCCATTGATGA
AGGCTCCATTCACTAGAGAAGACGTTAAGCACAGATTGGTTGGTCACTGGGGTACTACTCCAGGTTTGAACT
TCTTGATCGGTCACATCAACAGATTCATCGCTGACCACGGTCAAAACACTGTTATCATCATGGGTCCAGGTC
ACGGTGGTCCAGCTGGTACTTCTCAATCTTACTTGGACGGTACTTACACTGAAACTTTCCCAAAGATCACTAA
GGACGAAGCTGGTTTGCAAAAGTTCTTCAGACAATTCTCTTACCCAGGTGGTATCCCATCTCACTTCGCTCCA
GAAACTCCAGGTTCTATCCACGAAGGTGGTGAATTGGGTTACGCTTTGTCTCACGCTTACGGTGCTATCATGG
ACAACCCATCTTTGTTCGTTCCAGCTATCGTTGGTGACGGTGAAGCTGAAACTGGTCCATTGGCTACTGGTTG
GCAATCTAACAAGTTGGTTAACCCAAGAACTGACGGTATCGTTTTGCCAATCTTGCACTTGAACGGTTACAA
GATCGCTAACCCAACTATCTTGTCTCGTATCTCTGACGAAGAATTGCACGAATTTTTCCACGGTATGGGTTAC
GAACCATACGAATTTGTTGCTGGTTTCGACGACGAAGACCACATGTCTATCCACAGAAGATTCGCTGAATTG
TGGGAAACTATCTGGGACGAAATCTGTGACATCAAGGCTGCTGCTCAAACTGACAACGTTCACAGACCATT
CTACCCAATGTTGATCTTCAGAACTCCAAAGGGTTGGACTTGTCCAAAGTACATCGACGGTAAGAAGACTG
AAGGTTCTTGGAGAGCTCACCAAGTTCCATTGGCTTCTGCTAGAGACACTGAAGCTCACTTCGAAGTTTTGA
AGAACTGGTTGGAATCTTACAAGCCAGAAGAATTGTTCGACGCTAACGGTGCTGTTAAGGACGACGTTTTGG
CTTTCATGCCAAAGGGTGAATTGAGAATCGGTGCTAACCCAAACGCTAACGGTGGTGTTATCAGAGACGACT
TGAAGTTGCCAAACTTGGAAGACTACGAAGTTAAGGAAGTTGCTGAATACGGTCACGGTTGGGGTCAATTG
GAAGCTACTAGAACTTTGGGTGCTTACACTAGAGACATCATCAGAAACAACCCAAGAGACTTCAGAATCTT
CGGTCCAGACGAAACTGCTTCTAACAGATTGCAAGCTAGTTACGAAGTTACTAACAAGCAATGGGACGCTG
GTTACATCTCTGACGAAGTTGACGAACACATGCACGTTTCTGGTCAAGTTGTTGAACAATTGTCTGAACACC
AAATGGAAGGTTTCTTGGAAGCGTACTTGTTGACTGGTAGACACGGTATCTGGTCTTCTTACGAATCTTTCGT
TCACGTTATCGACTCTATGTTGAACCAACACGCTAAGTGGTTGGAAGCTACTGTTAGAGAAATCCCCTGGAG
AAAGCCAATCGCTTCTATGAACTTGTTGGTTTCTTCTCACGTTTGGAGACAAGACCACAACGGTTTCTCTCAC
CAAGACCCAGGTGTTACTTCTGTTTTGTTGAACAAGTGTTTCCACAACGACCACGTTATCGGTATCTACTTCG
CTACTGACGCTAACATGTTGTTGGCTATCGCTGAAAAGTGTTACAAGTCTACTAACAAGATCAACGCTATCAT
CGCTGGTAAGCAACCAGCTGCTACTTGGTTGACTTTGGACGAAGCTAGAGCTGAATTGGCTAAGGGTGCTGC
TGCTTGGGACTGGGCTTCTACTGCTAAGAACAACGACGAAGCTGAAGTTGTTTTGGCTGCTGCTGGTGACGT
TCCAACTCAAGAAATCATGGCTGCTTCTGACAAGTTGAAGGAATTGGGTGTTAAGTTCAAGGTTGTTAACGT
TGCTGACTTGTTGTCTTTGCAATCTGCTAAGGAAAACGACGAAGCTCTCTCTGACGAAGAATTTGCTGACAT
CTTCACTGCTGACAAGCCAGTTTTGTTCGCTTACCACTCTTACGCTCACGACGTTAGAGGTTTGATCTACGAC
AGACCAAACCACGACAACTTCAACGTTCACGGTTACGAAGAAGAAGGTTCTACTACTACTCCATACGACAT
GGTTAGAGTTAACAGAATCGACAGATACGAATTGACTGCTGAAGCTCTCAGAATGATCGACGCTGACAAGT
ACGCTGACAAGATCGACGAATTGGAAAAGTTCAGAGACGAAGCGTTCCAATTCGCTGTTGACAAGGGTTAC
GACCACCCAGACTACACTGACTGGGTTTACTCTGGTGTTAACACTGACAAGAAGGGTGCTGTTACTGCTACT
GCTGCTACTGCTGGTGACAACGAATAA

LjIFS ATGTTGGTTGAAATCGCTTTGGCCTTGCTGGCTTTAGCTTTATTCCTGCAATTCAGACCAACTCCCACCGCTAA

GTCTAAAGCTCTAAGACATTTGCCAAACCCACCCTCTCCAAAACCAAGATTACCATTCGTCGGCCATCTGCA
TTTGTTGGATCAACCATTGTTGCATAACTCTTTGATCAAGTTGGGTGAAAAGTACGGTCCCTTGTACACTTTGT
ACTTCGGTTCTATGCCAACTGTTGTTGCCTCTACTCCAGAATTGTTCAAGCTGTTCTTGCAAACTCATGAAGCT
ACTTCTTTCTCTACTAGATTCCAAACTTCTGCTATCAGAAGATTGACTTACGATAACTCTGTTGCTATGGTTCC
ATTCGCCCCATATTGGAAATTCATCAGGAAGGTCATCATGAACGATTTGTTGAACGCTACTACTGTTAACAAG
TTGAGACCATTGAGATCACAAGAAATCAGAAAGGTTTTGAAGGCTATGGCTCAATCTGCCGAATCTCAAAA
GCCATTGAACGTCACAGAAGAATTGTTGAAGTGGACTAACTCTACTATCTCTAGGATGATGTTGGGTGAAGC
CGAACATGTTAAGGACATCGTTAGGGAAGTTTTGAAGATTTTTGGTGAATACTCTTTGACTGATTTCATCTGG
CCATTGAAGAAGTTGAGAGTTGGCCAATACGAAAAGAGAATCGATGAAATCTTCAACAAGTTCGATCCAGT
TATCGAAAAGGTTATCAAGAAGAGACAAGAAATCATCAAGAGAAGAAAGGAAAGAAACGGTGAATTGGA
AGAAGGTGAACAATCTGTTGTTTTCTTGGATACTTTGTTGGAATACGCTGCTGATGAAAACATGGAAATCAA
GATCACTAAGGAACAAATCAAGGGTTTGGTTGTTGATTTCTTCTCTGCTGGCACCGATTCTACTGCTGTTGCT
ACTGATTGGGCTTTGGCTGAATTGATCAACAACCCAAGGGTCTTGAAGAAGGCTAGAGAAGAAGTCGACTC
CGTTGTTGGTAAGGATAGATTGGTCGACGAGTCTGATATCCAGAACTTGCCATACATCAGAGCCATCGTTAAG
GAGACATTCAGGATGCATCCCCCATTACCAGTTGTTAAGAGGAAGTGTGTTCAAGAATGTGAATTGAACGGT
TACGTTATCCCAGAAGGCGCTTTAGTTCTGTTCAACGTTTGGGCTGTCCAGAGAGATCCAAAATACTGGAAG
ACCCCACTGGAATTTAGACCAGAAAGGTTCCTGGAGGAGGCTGATATCGATTTAAAGGGTCAGCACTTCGA
ATTGTTGCCCTTTGGTTCTGGTAGGAGAATGTGTCCAGGTGTTAACTTGGCTACCGCTGGTATGGCTACTTTAT
TGTCTTCCGTCATCCAATGTTTCGAATTGCAAGTTGTTGGTCCAAAGGGCCAAATCTTGAAGGGTTCTGATGC
TAAGGCCAACATGGAAGAAAGGGCTGGTTTAACTGTTCCCAGAGCTAATTCCCTGATGTGTGTTCCACTGGC
TAGATCAAAGGTCGCTGCTGAATTACTGTCCTCTTAA

TpIFS ATGTTGTTGGAAATTGCTGTTGCTTTGTTGGTTATTGCTTTGTTTATTTACTTGAGACCAACTCCTACTGCTAAG
TCCAAGGCTTTGAGACATTTGCCAAATCCACCATCTCCAAAACCAAGATTGCCATTTATTGGTCATTTACATT
TGTTGGATCATCCATTGTTGCATATTTCCTTAATTAGATTGGGTGAAAGATACGGTCCATTGTATTCTTTGTATTT
CGGTTCTATGCCAACCGTTGTTGCTTCTACTCCTGATTTGTTTAAGTTGTTTTTGCAAACTCATGAAGCTACTTC
TTTTAATACTAGATTCCAAACTTCCGCTATTAGAAGATTGACTTATGATAATTCCGTTGCTATGGTTCCTTTTGG
TCCATATTGGAAATTTGTTAGAAAGTTGATTATGAACGATTTGTTGAACGCAACTACCGTTAACAAATTGAGA
CCATTGAGATCTAAAGAAATTAGAAAGGTTTTGAACGTTATGGCTAATTCTGCTGAAACTCAACAACCATTG
AACATTACAGTTGAATTGTTGAAATGGACAAACAGTACTATTTCAACCATGATGTTGGGTGAAGCAGAAGAA
GTTAGAGATATTGCTAGAGATGTTTTGAAAATTTTCGGTGAATACTCAGTTACTGATTTTATTGGTCCATTGAA
GATTTTCAAGAAGTTTGGTAATTACGAACAAAGAATTGATGCTATTTTCAACAAGTACGATCCAATTATTGAA
AGAGTTATTAAGAAGAGACAAGGTATTGTTAACAAGAGAAAAAATGGTGAAGTTTTGGTTGGTGAAGAAGA
AAATGTTGTTTTTTTGGATACTTTGTTGGAATTTGCTCAAGATGAAACTATGGAAATTAAAATTACTAAAGAA
CAAATTAAAGGTTTGGTTGATTTTTTTTCTGCTGGTACTGATTCTACTGCTGTTGCTACTGAATGGACTTTGGCT
GAATTGATTAATAATCCAAGAGTTTTGAAAAAAGCTAGAGAAGAAGTTGAATCTGTTGTTGGTAAAGATAGA
TTGGTTGATGAATCTGATATTCAAAATTTGCCATATATTAGAGCTATGGTTAAAGAAGTTTTTAGATTGCATCC
ACCATTGCCAGTTGTTAAAAGAAAATGTACTGAAGAATGTGAAATTAATGGTTATGTTATTCCAGAAGGTGCT
TTGATTTTGTTTAATGTTTGGCAAGTTGGTAGAGATCCAAAATATTGGGAAAAACCATTGGAATTTAGACCAG
AAAGATTTTTGGAAAATGCTGGTGTTGGTGAAGGTGAAGCTTCTTCTATTGATTTGAGAGGTCAACATTTTAC
TTTGTTGCCATTTGGTTCTGGTAGAAGAATGTGTCCAGGTGTTAATTTGGCTACTGCTGGTATGGCTACTTTGT
TGTCTTCTATTATTCAATGTTTTGATTTGCAAGTTCCAGGTCCAAATGGTCAAATTTTGAAAGGTTCTGATGCT
AAAGTTACTATGGATGAAAGACCAGGTTTGTCTGTTCCAAGAGCTCAAAATTTGGTTTGTGTTCCATTGGCTA
GAGCTGGTGCTGCTAAATTGTTGTCTTCTTAA

GmCPR ATGCAGGACTCTGGTTCTATGAAGATCAGCCCATTGGACTTGATGTCCGCTATTATCAAGGGCAAGCTGGAC

CCTTCTAACGTTTCTTCTTCCTCTTCCAACGCCGCTGCTGCTGTCCTGCTGGAGAACAGGCAGTTCTTAATGTT
GCTGACCACCTCTGTCGCTGTTTTAGTTGGTTGTTTCGTCGTCTTCATCTGGAGAAGATCCTCTTCTCCAAAGG
CTAAGCCATTGGAACCACCAAAAAGGGTTATCGAGAAGCTGCCAGAAATCGAAGTCGATGATGGTACTAAG
AAGGTCACCATCTTCTTCGGTACGCAAACAGGTACTGCTGAAGGTTTTGCTAAGGCTATCGCTGAAGAGGCT
AAGGCTAGATATGACAAGGCTACATTCAGGGTCGTCGATATGGATGATTACGCTGCTGATGACGACGAATAC
GAAGAAAGATTCAAGAAGGAGACGCACGCTTTGTTCTTCTTGGCTACTTACGGTGACGGTGAACCAACTGA
TAACGCTGCTAGATTCTACAAGTGGTTCACCGAAGGTGGTGAAAAGGGTGAAGGTTGGTTAGAAAACCTGC
ACTACGGTGTTTTCGGCTTGGGTAATAGACAGTACGAGCATTTCAACAAGGTCGCTAAGGTCGTTGACGATA
TGTTGGCTGAACAGGGTGGTAAAAGGTTGGTTCCAGTTGGTTTGGGTGACGATGATCAATGTATCGAGGACG
ATTTCACCGCTTGGAAAGAAGAACTGTGGCCAGCTTTGGATGACTTATTGAGAGACGAGGACGACACAACT
GTCTCTACTCCATATACCGCTGCTGTTTTGGAGTACAGAGTCGTTATCCACGACCCATTAGAGGCTTCTGTTGA
TGAAAAGAAGTGGCACAACGTCAACGGTCATGCTATTGTTGACGCTCAACACCCAGTTAGAGCTAATGTTG
CTGTCAGAAAGGAGCTGCATACCCCAGCTTCTGATAGATCATGTACCCATCTGGAGTTCGACATCTCTGGTAC
AGGTGTTACTTACGAGACGGGTGATCATGTTGGTGTTTACTGTGAGAACCTGTCCGAAACAGTCGAAGAAGC
TATCAGACTGATCGGTTTGTCCCCAGATACTTACTTCTCCATCCACACTGACGACGAAGATGGTAAACCATTG
GGTGGTTCTTCCTTGCCACCAACTTTTCCACCATGTACACTGAGAAAGGCCTTGGCTCAATACGCTGATGTTT
TGTCTTCCCCAAAGAAGTCCGCTTTGTTGGCTTTGGCTGCTCATGCTTCTGATCCATCTGAAGCTGATAGGTTG
AGGCATTTGGCTTCTCCAGCTGGTAAAGATGAGTACTCTGAGTGGGTTATCACCTCTCAAAGGTCTTTGCTGG
AGGTTATGGCTGAATTTCCCTCTGCTAAGCCACCAATTGGTGTTTTTTTCGCCGCTGTTGCTCCAAGATTGCAA
CCAAGATTCTACTCCATCTCCTCCTCTCCAAGAATGGTTCCAAACAGAATCCACGTCACCTGTGCTTTGGTTC
ATGATAAGATGCCCACCGGTAGAATCCATAAGGGTGTTTGTTCCACCTGGATGAAGAACTCTGTCCCATTGG
AAAAGTCCCAGGATTGTTCTTGGGCTCCAATTTTCGTCAGGACTTCTAACTTCAGGCTGCCAGCTGATAACA
AGGTTCCAATTATCATGATCGGTCCAGGCACTGGTTTGGCTCCATTTAGAGGTTTTCTGCAGGAGAGACTGGC
TTTAAAGGGTGGTGGTGCTGAATTGGGTCCATCTGTTTTGTTCTTCGGCTGTAGAAACAGGCAGATGGATTAC
ATCTACGAGGACGAACTGAACCACTTCGTTAACACAGGTGCTTTGTCCGAACTGATCTTGGCTTTTTCCAGG
GAAGGTCCAACAAAGGAATACGTTCAGCACAAGATGATGGAGAAGGCTTCTGAAATCTGGTCCATGATCTC
CCAAGGTGCTTATATCTACGTCTGCGGTGATGCTAAGGGTATGGCTAGAGATGTTCACAGAGCTTTGCACACA
ATCCTGCAAGAACAGGGTTCTTTGGACTCTTCTAAGGCTGAATCTATGGTTAAGAACCTGCAGACCACCGGT
AGATATTTGAGAGATGTCTGGTAA

LjCPR ATGGAGGAATCCTCTTCTATGAAGATCAGTCCCCTGGATCTGATGTCTGCTATGATTAAGGGCACCTTGGACC
CATCTAACGTTTCTTCTACCTCCGGTGCTGGTTCTGTTTTTTTGGAAAACAGAGAATTTGTTATGGTTTTGACT
ACTTCTATCGCTGTTTTGATCGGTTGTGTTGTTGTTTTCATCTGGAGAAGATCAACTGGTAACAAGGCCAAGT
CCATCGAACCACCAAAAAGAGTTGTTGAAAAGTTGTCTGATGAAGCTGAAGTTGATGATGGTACTAGAAAG
GTTACTATCTTCTTCGGTACTCAAACTGGTACTGCCGAAGGTTTTGCTAAGGCTATTGCTGAAGAAGCCAAGG
TTAGGTACGAGAAAGCTAAGTTCAAGATCGTTGATATGGATGATTACGCTCAAGATGATGATGAATACGAAG
AAAAGTTGAAGAAGGAAACTTTGGCTTTGTTCTTCTTGGCTACTTACGGCGATGGTGAACCAACTGATAACG
CTGCTAGATTCTACAAGTGGTTCCTGGAAGGTGACGAAAAGGAAGAAGGTTGGTTGAGAAACTTGGAATAC
GCCGTTTTCGGCTTGGGTAATAGACAATACGAACATTTCAACAAGGTTGCTATCGAAGTTGATGACAAGTTG
GCCGATTTCGGTGGTAAAAGACTGGTTAAGGTTGGCTTGGGTGACGATGATCAATGTATCGAAGATGATTTC
ACTGCTTGGAAGGAAGAATTGTGGCCAGCTTTGGATGAGTTGTTGAGAGGTGATGACGACACTACAGTCTCT
ACACCATACACTGCTGCTGTTTTGGAGTACAGAGTCGTTATCCACGACCCATTAGACGCTTCTGTTGATGAAA
AGAAGTGGCACAACGTTAACGGCCATGCTATTGTTGACGCTCAACATCCAGTCAGATCCAATGTTGCTGTCA
GAAAGGAGCTGCATACCCCAGTTTCTGATAGATCATGCACCCATCTGGAGTTCGATATCTCTGGTACTGGTGT
TGCTTACGAGACTGGTGATCATGTTGGTGTTTACTGCGAGAACCTGTCTGAAACAGTCGAAGAAGCTGTCAG

	ACTGTTGGGTTTATCCCCAGATACTTACTTCTCTGTTCATACTGATGATGAAGATGGTAAGCCATTGTCTGGCT<br>CTTCTTTGCCACCAACTTTCCCACCATGTACTTTGAGAACCGCTATCGCTAGATACGCTGATGTTTTGTCCTCC<br>CCAAAGAAGTCTGTCTTGTTGGCTTTGGCTGCTCATGCTTCTAACCCATCTGAAGCTGATAGGTTGAGGCATT<br>TGGCTTCTCCAGCTGGTAAAGATGAGTACTCTGAGTGGGTTATCGCCTCTCAAAGATCATTGCTGGAGGTTAT<br>GGCCGAATTTCCATCTGCTAAGCCACCAATTGGCGTTTTTTTCGCTGCTATCGCTCCAAGACTGCAACCAAGA<br>TTTTACTCCATCTCTTCTTCTCCAAGAATGGCCCCATCTAGGATTCATGTTACCTGTGCTCTGGTCAACGACAA<br>AATGCCAACAGGTAGAATCCATAGAGGTGTCTGTTCCACATGGATGAAGAACTCTGTCCCATTGGAGAAGTC<br>CCAAGATTGTTCTTGGGCTCCAATCTTCGTCAGACAATCTAACTTCAAGTTGCCAGCCGACAACAAGGTTCC<br>AATTATCATGATCGGTCCAGGCACTGGTTTGGCTCCATTCAGAGGTTTTCTGCAAGAGAGACTGGCTTTGAA<br>GGAGGATGGTGCTGAATTGGGTCCATCTGTTTTGTTCTTCGGCTGTAGAAACAGACAAATGGATTACATCTAC<br>GAAGATGAATTGAACCATTTCGTTAACTCTGGTGCTTTGTCTGAATTGATCGTTGCCTTCTCCAGGGAAGGTC<br>CAACTAAAGAATACGTCCAGCACAAGATGATGGAAAAGGCTTCTGACATCTGGAACATGATCTCCCAAGGT<br>GCTTACATCTACGTCTGTGGTGATGCTAAGGGTATGGCTAGAGATGTTCACAGAACCTTGCACACTATCCTGC<br>AAGAACAGGGTTCTTTGGACTCTTCTAAGGCTGAAGGTATGGTCAAGAACCTGCAATTGAACGGTAGGTACT<br>TGAGGGATGTCTGGTAA
GmHID	ATGGCTAAGGAGATCGTCAAGGAACTGTTGCCATTGATCAGGGTTTACAAGGACGGTTCTGTCGAAAGACT<br>GTTGTCTTCTGAGAACGTCGCTGCTTCTCCAGAAGATCCACAAACTGGTGTTTCCTCTAAGGACATCGTCATC<br>GCTGATAACCCATACGTTTCCGCTAGAATCTTCCTGCCAAAGTCTCATCACACCAACAACAAGCTGCCAATC<br>TTCTTGTACTTCCACGGTGGTGCTTTCTGTGTTGAATCTGCTTTCTCCTTCTTCGTTCATAGATACTTGAACATC<br>TTGGCTTCTGAAGCTAACATCATCGCCATCTCTGTCGATTTCAGGTTGTTGCCACACCATCCAATCCCAGCTG<br>CTTATGAAGATGGTTGGACTACACTGAAGTGGATCGCTTCTCATGCTAACAACACCAACACCACAAACCCA<br>GAACCTTGGTTACTGAACCACGCTGATTTCACTAAGGTTTACGTTGGCGGTGAGACATCTGGTGCTAATATTG<br>CTCACAACTTGTTGTTGAGAGCTGGCAATGAGTCCTTGCCAGGTGATTTAAAGATCCTGGGTGGTTTGCTGTG<br>CTGTCCATTTTTTTGGGGTTCCAAGCCAATCGGTTCCGAAGCTGTTGAAGGTCATGAACAATCCCTGGCTATG<br>AAGGTTTGGAACTTCGCTTGTCCAGACGCTCCAGGTGGTATCGATAATCCTTGGATTAACCCCTGCGTCCCAG<br>GTGCTCCATCTTTGGCTACTTTAGCTTGTTCCAAGTTGTTGGTTACTATCACTGGTAAGGATGAATTTAGAGAT<br>AGAGATATCTTGTACCATCATACTGTTGAACAATCTGGTTGGCAAGGCGAATTGCAATTGTTCGATGCTGGTG<br>ATGAAGAACATGCCTTCCAATTGTTCAAGCCCGAAACTCATTTGGCCAAGGCTATGATTAAGAGATTGGCCT<br>CTTTCTTGGTTTAA

Table S3. The main primers used in this study.

Primer	Sequence (5′-3′)
P_{SED1}-pRS426-F	ATTCCTGCAGCCCGGGGGATCCACTAGTTCGGACAAAAAGGCATATATCGCTAAAA
P_{SED1}-R	CTTAATAGAGCGAACGTATTTTATTTTG
EbCHS-P_{SED1}-F	AGCAAAATAAAATACGTTCGCTCTATTAAGATGGCTTCTTCTATCGACATCG
EbCHS-T_{ENO2}-R	AAAGACTAATAATTCTTAGTTAAAAGCACTTTAAGTAGCGATAGCAGTAGTAGTT
SbCHS-P_{SED1}-F	AGCAAAATAAAATACGTTCGCTCTATTAAGATGGCTGCGGCGACCGTTACC
SbCHS-T_{ENO2}-R	AATTCTTAGTTAAAAGCACTTTACGCGGTAATCGCCGCACCGGTGGT
PhCHS-P_{SED1}-F	AGCAAAATAAAATACGTTCGCTCTATTAAGATGGTTACCGTGGAAGAATATCG
PhCHS-T_{ENO2}-R	AAGACTAATAATTCTTAGTTAAAAGCACTTTAGGTTGCAACGCTATGCAGAACAA
HaCHS-P_{SED1}-F	AAAATAAAATACGTTCGCTCTATTAAGATGATGGTTACCGTGGAGGAAGT
HaCHS-T_{ENO2}-R	AAAGACTAATAATTCTTAGTTAAAAGCACTTTAGTTGATCGCCACGCTGTG
AsCHS-P_{SED1}-F	TAAAATACGTTCGCTCTATTAAGATGGCAGCCAAAGTTGAAGA
AcCHS-T_{ENO2}-R	AATAATTCTTAGTTAAAAGCACTTTAATGTGCGCTTTCGGTTGCAA
GmCHS-P_{SED1}-F	AGCAAAATAAAATACGTTCGCTCTATTAAGATGGTTAGCGTGGCGGAGATT
GmCHS-T_{ENO2}-R	AAAGACTAATAATTCTTAGTTAAAAGCACTTTAAATCGCCACGCTGTGCA
PpCHS-P_{SED1}-F	CAAAATAAAATACGTTCGCTCTATTAAGATGGCGCCGCTGGTGAAAAACG
PpCHS-T_{ENO2}-R	AGACTAATAATTCTTAGTTAAAAGCACTTTAGCAACGGATGCTCTCGCTAC
MdCHS-P_{SED1}-F	AGCAAAATAAAATACGTTCGCTCTATTAAGATGGTTACCGTGGAGGAAGT
MdCHS-T_{ENO2}-R	AAAGACTAATAATTCTTAGTTAAAAGCACTTTACGCCGCCACGCTGTGCAG
T_{ENO2}-F	AGTGCTTTTAACTAAGAATTATTAGTCTT
T_{ENO2}-P_{TEF1}-R	GTAGAAACATTTTGAAGCTATGGTGTGTGGAGGTATCATCTCCATCTCCCA
P_{TEF1}-F	CCACACACCATAGCTTCAAAAT
P_{TEF1}-R	CTTAGATTAGATTGCTATGCTTTCTTTCTA
Ha4CL-P_{TEF1}-F	GCATAGCAATCTAATCTAAGATGGCTCCAGAAAAGGAAATCAT
Ha4CL-R	TCAGTTTGGGACACCGGCGG
T_{GPM1}-Ha4CL-F	GCTAAATTAGCCGCCGGTGTCCCAAACTGAGTCTGAAGAATGAATGATTTGATGATT
T_{GPM1}-R	TATTCGAACTGCCCATTCAGC
P_{TEF2}-T_{GPM1}-F	CTGAATGGGCAGTTCGAATAGGGCGCCATAACCAAGGT

P_{TEF2}-ErCHI-R	TCTCATTGGTTCGAACTTGAAATCAGCCATGTTTAGTTAATTATAGTTCGTTGACCG
ErCHI-F	ATGGCTGATTTCAAGTTCGA
ErCHI-T_{GPD}-R	ATTTAAATGCAAGATTTAAAGTAAATTCACTTATCTCATGGTAATGTAACCTCTGT
T_{GPD}-F	GTGAATTTACTTTAAATCTTGCATTT
T_{GPD}-pRS426-R	CACTAAAGGGAACAAAAGCTGGAGCTCCACGGAATCTGTGTATATTACTGCATCT
pRS426-F	GTGGAGCTCCAGCTTTTGTTC
pRS426-R	GAACTAGTGGATCCCCCGG
CUT-F	AGGGTTTTCCCAGTCACG
CUT-R	AGTTACCTCACTCATTAGGCAC
YPRC*Δ*15-UP-F	TGTCCTGCAAATCGTGTAAATA
YPRC*Δ*15-UP-loxp1-R	ACTGGCCGTCGTTTTACATTTGCGAAACCCTATGCTCTGTTGT
loxp1-F	TGTAAAACGACGGCCAGT
loxp1-R	AGGAGGCCAAGAGTAATAGAAAA
LEU2-loxp1-F	TCTATTACTCTTGGCCTCCTTTAAGCAAGGATTTTCTTAA
LEU2-loxp2-R	GCTTTAAATAATCGGTGTCAAACTGTGGGAATACTCAGGT
loxp2-F	TGACACCGATTATTTAAAGCTGC
loxp2-R	ATTCGAGCTCGGTACCCGGG
T_{ADH1}-loxp2-R	CCCGGGTACCGAGCTCGAATGAGCGACCTCATGCTATACCT
P_{HXT7}-P_{TEF1}-F	ATTTTGAAGCTATGGTGTGTGGACTTCTCGTAGGAACAATTTC
P_{HXT7}-R	TTTTTGATTAAAATTAAAAAAACTTTTTGTTTTTGTGTTTATTCTTTGTTCTTAG
EbCHS-P_{HXT7}-F	TTTTAATTTTAATCAAAAAATGGCTTCTTCTATCGACATCG
T_{CYC1}-ErCHI-F	AACTACAGAGGTTACATTACCATGAGATAAATCCGCTCTAACCGAAAAGG
YPRC*Δ*15-DOWN-T_{CYC1}-F	AAGGTTTTGGGACGCTCGAAGAATGGAAGGTCGGGATGAGCA
YPRC*Δ*15-DOWN-R	TTATTAAAGCTTGATAAATTACTGAAATTCCAC
YPRC*Δ*15-UP-YZ-F	ATTACTATCCGATAACGCCAG
YPRC*Δ*15-DOWN-YZ-R	TTTTGGACATCTATGAAACACCC
P_{PGK1}-426-F	ATTCCTGCAGCCCGGGGGATCCACTAGTTCTGTTTGCAAAAAGAACAAAACTGA
P_{PGK1}-R	TGTTTTATATTTGTTGTAAAAAGTAGA
SbCHIL-P_{PGK1}-F	TTATCTACTTTTTACAACAAATATAAAACAATGTTGATCTCCGCTGTCGG

SbCHIL-T$_{GPD}$-R	ATTTAAATGCAAGATTTAAAGTAAATTCACTCAGGCGGCGAGCAAAGCAG
PhCHIL-P$_{PGK1}$-F	TTATCTACTTTTTACAACAAATATAAAACAATGGGTAAGAACGAAGTTATGGTT
PhCHIL-T$_{GPD}$-R	ATTTAAATGCAAGATTTAAAGTAAATTCACTTACTTGGACAATTCAGCGGACA
GmCHIL-P$_{PGK1}$-F	ATTATCTACTTTTTACAACAAATATAAAACAATGGCGACCGAGGAAGTGC
GmCHIL-T$_{GPD}$-R	ATTTAAATGCAAGATTTAAAGTAAATTCACTTATTTGCTCAGTTCTTGGCTAAAG
AtCHIL-P$_{PGK1}$-F	TTATCTACTTTTTACAACAAATATAAAACAATGGGCACCGAAATGGTTATG
AtCHIL-T$_{GPD}$-R	ATTTAAATGCAAGATTTAAAGTAAATTCACTTAGGTCAGAACCGCGCTGAT
MdCHIL-P$_{PGK1}$-F	TTATCTACTTTTTACAACAAATATAAAACAATGGGCACCGAGGTGGTTCTG
MdCHIL-T$_{GPD}$-R	ATTTAAATGCAAGATTTAAAGTAAATTCACTTATTTGGTCAGCTCCGCGC
P$_{PGK1}$-T$_{CYC1}$-F	TTGCTTGAGAAGGTTTTGGGACGCTCGAAGTGTTTGCAAAAAGAACAAAACTGAAAA
YPRCΔ15-T$_{GPD}$-F	TATCTAGATGCAGTAATATACACAGATTCCAATGGAAGGTCGGGATGAGCATATAC
MLS-EbCHS-F	TTTTTAATTTTAATCAAAAAATGCTTTCACTACGTCAATCTATAAGATTTTTCAAGCCAGCCACAAGAACTTTGTGTAGCTCTAGATATCTGCTTCAGGCTTCTTCTATCGACATCGC
MLS-Ha4CL-F	GAAAGCATAGCAATCTAATCTAAGATGCTTTCACTACGTCAATCTATAAGATTTTTCAAGCCAGCCACAAGAACTTTGTGTAGCTCTAGATATCTGCTTCAGGCTCCAGAAAAGGAAATCATCTTC
MLS-ErCHI-F	AATATACGGTCAACGAACTATAATTAACTAAACATGCTTTCACTACGTCAATCTATAAGATTTTTCAAGCCAGCCACAAGAACTTTGTGTAGCTCTAGATATCTGCTTCAGGCTGATTTCAAGTTCGAACCAATGAG
MLS-SbCHIL-F	TTTACAACAAATATAAAACAATGCTTTCACTACGTCAATCTATAAGATTTTTCAAGCCAGCCACAAGAACTTTGTGTAGCTCTAGATATCTGCTTCAGTTGATCTCCGCTGTCGGCTCTGAAAC
COXIV-F	ATGCTTTCACTACGTCAATCT
COXIV-mCherry-linker-R	TTTACTAACAGAACCACCACCACCAGAACCACCACCACCAGAACCACCACCACCGTGATGGTGGTCATCATTTGG
mCherry-linker-F	GGTGGTGGTGGTTCTGGTGGTGGTGGTTCTGGTGGTGGTGGTTCTGTTAGTAAAGGAGAAGAAAATAACATGG
mCherry-T$_{ACS1}$-R	GTCAATATAAAAAGGAAAGAAATCATCATTATGCGGTACCAGAACCTTTG
Ha4CL-linker-EGFP-R	ACCTTTAGAAGAACCACCACCACCAGAACCACCACCACCAGAACCACCACCACCGTTTGGGACACCGGCGGC
EGFP-linker-F	GGTGGTGGTGGTTCTGGTGGTGGTGGTTCTGGTGGTGGTGGTTCTTCTAAAGGTGAAGAATTATTCACTGGT
EGFP-T$_{ACS1}$-R	TATAAAAAGGAAAGAAATCATCATCATTTGTACAATTCATCCATACCATG
pRS416-F	ACCGCGGTGGAGCTCCAGCT
pRS416-R	ACTAGTGGATCCCCCGGGCT

PEX3-P$_{TDH3}$-F	GAATAAACACACATAAACAAACAAAATGGCCCCAAATCAAAGATCA
PEX3-mCherry-linker-R	CTTTACTAACAGAACCACCACCACCAGAACCACCACCACCAGAACCACCACCACCAGGCTTGAAGGAAAACGAGC
T$_{ADH1}$-ePTS1-F	TTGGGAAGAGGTAGAAGATCCAAATTGTAAGCGAATTTCTTATGATTTATGATTTTT
EbCHS-ePTS1-R	TTACAATTTGGATCTTCTACCTCTTCCCAAAGTAGCGATAGCAGTAGTAGT
Ha4CL-ePTS1-R	TCACAATTTGGATCTTCTACCTCTTCCCAAGTTTGGGACACCGGCGGCTA
T$_{GPM1}$-ePTS1-F	AACTTGGGAAGAGGTAGAAGATCCAAATTGTGAGTCTGAAGAATGAATGATTTGATGA
ErCHI-ePTS1-R	TTACAATTTGGATCTTCTACCTCTTCCCAATCTCATGGTAATGTAACCTCTGTAG
T$_{CYC1}$-ePTS1-F	GATTGGGAAGAGGTAGAAGATCCAAATTGTAAATCCGCTCTAACCGAAAAGGAAGG
SbCHIL-ePTS1-R	TAAATGCAAGATTTAAAGTAAATTCACTCACAATTTGGATCTTCTACCTCTTCCCAAGGCGGCGAGCAAAGCAGCGAATC
EGFP-T$_{ACS1}$-ePTS1-R	AAAAAGGAAAGAAATCATCATCACAATTTGGATCTTCTACCTCTTCCCAATTTGTACAATTCATCCATACCATGG
Ha4CL-YZ-F	CCCACAAATTTCTGACGTTG
ErCHI-YZ-F	TTGTTCGAAAAGGTTGTTCCAAT
HO-UP-F	ATGCTTTCTGAAAACACGACT
HO-UP-loxp1-R	TGCAAGCTTGGCACTGGCCGTCGTTTTACACTAACTTCAATGTCAAAGTTGAAATAT
Trp1-loxp1-F	TTTTTCTATTACTCTTGGCCTCCTGAGAGTGCACCATAAACGACA
Trp1-loxp2-R	GCAGCTTTAAATAATCGGTGTCACTATTTCTTAGCATTTTTGAC
P$_{TDH1}$-loxp2-F	ACGAAGTTATCCCGGGTACCGAGCTCGAATGGAATAGGATATGCGACGAAGAC
P$_{TDH1}$-mACC1-R	AGAAGACTCGAATAAGCTTTCTTCGCTCATTTTGTTTTGTGTGTAAATTTAGTGAAG
mACC1-F	ATGAGCGAAGAAAGCTTATTCG
mACC1-S659A-F	ACTGCGTCAACTAGCTGATGGT
mACC1-S659A-R	CCATCAGCTAGTTGACGCAG
mACC1-S686A-F	TGCTGCTACAAGATTAGCCGTTGACT
mACC1-S686A-R	GTAGTCATAGAGTCAACGGCTAATCTTGTA
mACC1-S1157A-F	GTATGAACAGGGCTGTTGCTGTTTCAGATTTGTCATATGT
mACC1-S1157A-R	ACATATGACAAATCTGAAACAGCAACAGCCCTGTTC
mACC1-ePTS1-R	TTACAATTTGGATCTTCTACCTCTTCCCAATTTCAAAGTCTTCAACAATTTTTCTTTATC
T$_{ACS1}$-mACC1-F	AGACTTTGAAATTGGGAAGAGGTAGAAGATCCAAATTGTAATGATGATTTCTTTC

	CTTTTTATATTGACG
T_{ACS1}-HO-DOWN-R	ATACGGGTTCCCTTTTTATAATTGGCGGAATCGATACAAGGACTGCCCA
HO-DOWN-F	TTCCGCCAATTATAAAAAGGGAAC
HO-DOWN-R	TTAGCAGATGCGCGCACC
mACC1-YZ-F1	CCGCTACAAAGGCTTTCTTA
mACC1-YZ-F2	ACTTCAATTCCTAACCCATCAAG
HO-UP-YZ-F	CGCACTATTCATCATTAAATATTTAAAGC
HO-DOWN-YZ-R	AGAAAAAGTCTAAAAATGGTTTTTTTCAT
1622b-UP-F	AACATTTAAGTCACAAGGAGGAATAT
1622b-UP-loxp1-R	TGCAAGCTTGGCACTGGCCGTCGTTTTACAAACTACTTTTCTTAAACTGTCAACA GC
P_{HXT7}-P_{TDH3}-F	TATATTTAGTGGATGCCAGGAATAAACTGTACTTCTCGTAGGAACAATTTCG
FjTAL-P_{TDH3}-F	GTTTCGAATAAACACACATAAACAAACAAAATGAACACCATCAACGAGTACC
FjTAL-T_{ENO2}-R	AAAGACTAATAATTCTTAGTTAAAAGCACTTTAGTTGTTGATCAAATGGTCCTTG
1622b-DOWN-T_{ENO2}-F	TATGCATATGGGAGATGGAGATGATACCTGTAGATACTCGTCTTACGAAATTGGA
1622b-DOWN-R	ACTTTGGAAAAGAAGGTACGGAC
FjTAL-ePTS1-R	AAAGACTAATAATTCTTAGTTAAAAGCACTTTACAATTTGGATCTTCTACCTCTTC CCAAGTTGTTGATCAAATGGTCCTTGAC
1622b-UP-YZ-F	ATGTCTCCTTATCCTTGAGTGC
1622b-DOWN-YZ-R	AAATTTGTTCCCTCAGATGTGAC
PDC5-UP-F	TAGGCATAATCACCGAAGAAGAAT
PDC5-UP-loxp1-R	TGCAAGCTTGGCACTGGCCGTCGTTTTACATTTGTTCTTCTTGTTATTGTATTGTGT
PDC5-DOWN-loxp2-F	ACGAAGTTATCCCGGGTACCGAGCTCGAATGCTAATTAACATAAAACTCATGATT CAAC
PDC5-DOWN-R	TCTACTGGTGATTTTTCATCGAAC
PDC5-UP-YZ-F	AATGACGACGAGCCTGAAG
PDC5-DOWN-YZ-R	TCAATTGGCGAAGCAGAAC
ARO10-UP-F	GCAAGGTTAATTAATTAACATCTACCA
ARO10-UP-loxp1-R	GCAAGCTTGGCACTGGCCGTCGTTTTACAGCTTAAGGGAGTTTCTTTGTTA
ARO10-DOWN-loxp2-F	ACGAAGTTATCCCGGGTACCGAGCTCGAATAAACTTGTGGGCGCAATTAT
ARO10-DOWN-R	ATAGGGCCTCTTCTGTTTGGAA
ARO10-UP-YZ-F	CCTCTTCTTCTTGTGTGTTTAAG

ARO10-DOWN-YZ-R	GTCCAAGAGCTTGGCAAC
PHA2-UP-F	TTGCAGTTCCATTCTGATTTGGT
PHA2-UP-R	GAAGAACAATCCAATCTTATAAAGGTGA
loxp1-PHA2-UP-F	GGTCACCTTTATAAGATTGGATTGTTCTTCTGTAAAACGACGGCCAGTG
P_{TDH3}-loxp2-F	ACGAAGTTATCCCGGGTACCGAGCTCGAATACAGTTTATTCCTGGCATCCA
P_{TDH3}-R	TTTGTTTGTTTATGTGTGTTTATTCG
P_{TDH3}-ARO4-F	GTTTCGAATAAACACACATAAACAAACAAAATGAGTGAATCTCCAATGTTCG
ARO4-K229L-F	CACCATTTCATGGGTGTTACTTTGCATGGTGTTGCTGCTATCAC
ARO4-K229L-R	GTGATAGCAGCAACACCATGCAAAGTAACACCCATGAAATGGTG
T_{ADH1}-ARO4-R	AATAAAAATCATAAATCATAAGAAATTCGCCTATTTCTTGTTAACTTCTCTTCTTTG TC
T_{ADH1}-F	GCGAATTTCTTATGATTTATGATTTTTATT
P_{HXT7}-T_{ADH1}-R	GCTGCGGCTTTGTTTAAGGCAGAAACTAGACGTAAAAAAAGCATGCACGTAT
P_{HXT7}-ARO7-F	ACAAAAAGTTTTTTTAATTTTAATCAAAAAATGGATTTCACAAAACCAGAAAC
ARO7-G141S-F	GTGATGATAAGAATAACTTCAGTTCTGTTGCCACTAGAGATA
ARO7-G141S-R	TATCTCTAGTGGCAACAGAACTGAAGTTATTCTTATCATCAC
T_{TEF1}-ARO7-R	ATGCAACTAGAAAAGTCTTATCAATCTCCTTTACTCTTCCAACCTTCTTAGCAAGT AT
T_{TEF1}-F	AGGAGATTGATAAGACTTTTCTAGTTG
P_{HXK1}-T_{TEF1}-R	AGGAACACGTACTCCTTAAAAGCGTCTAAAACTGAAAAAAGAGGGGAATTTTTA GAT
P_{HXK1}-F	TTTAGACGCTTTTAAGGAGTACG
P_{HXK1}-R	CTTATTTTTTCAGTATTCTAATTGAGTTGTTT
P_{HXK1}-EcAROL-F	ACAACTCAATTAGAATACTGAAAAAATAAGATGACACAACCTCTTTTTCTGAT
T_{CYC1}-EcAROL-R	CTAACTCCTTCCTTTTCGGTTAGAGCGGATTCAACAATTGATCGTCTGTGC
T_{CYC1}-PHA2-DOWN-F	TTGCTTGAGAAGGTTTTGGGACGCTCGAAGCTGAATCAGTGAACCAATGCA
PHA2-DOWN-R	TTATTTGTGATAATATCTCTCATTTCTGGG
PHA2-UP-YZ-F	ACACATTGTATGATGACTTCCC
PHA2-DOWN-YZ-R	AAAGTGCGCTTTGACGAGAT
308a-UP-F	TATTTCAGAAAAATTATTCAAAACTAAGAAGAATGAG
308a-UP-loxp1-R	GCTTGGCACTGGCCGTCGTTTTACATTAGATAAAAAGAAAAAAATTCGAAGTTAA TGTTG
Bbxfpk-P_{TDH1}-F	TACTTCACTAAATTTACACACAAAACAAAATGACTTCTCCAGTTATCGGT

Bbxfpk-T_{GPD}-R	ATTTAAATGCAAGATTTAAAGTAAATTCACTTATTCGTTGTCACCAGCAGTAG
308a-T_{GPD}-R	AATAGAAGTGGTAGCAATATGTAGCAAAGAGGAATCTGTGTATATTACTGCATCTAG
308a-DOWN-F	TCTTTGCTACATATTGCTACCACT
308a-DOWN-R	TGATAGAACGAGTACAACACCCG
308a-UP-YZ-F	CAGCGAAGATAACGGTTACACA
308a-DOWN-YZ-R	AATCTAATTCCATTATTTCAGGTACTC
YORWΔ17-UP-F	TCCTCCCTATAGGATATAGTAATCCTC
YORWΔ17-UP-loxp1-R	TGCAAGCTTGGCACTGGCCGTCGTTTTACAGATGGCATGAGTTATGGTTGCAC
P_{TDH1}-R	TTTGTTTTGTGTGTAAATTTAGTGAAG
LjIFS-P_{TDH1}-F	GTACTTCACTAAATTTACACACAAAACAAAATGTTGGTTGAAATCGCTTTGG
LjIFS-T_{PGK1}-R	ATTGATCTATCGATTTCAATTCAATTCAATTTAAGAGGACAGTAATTCAGCAGC
LjIFS-YZ-F	CAAGTTCGATCCAGTTATCGAA
LjIFS-YZ-R	GTTCACCTTCTTCCAATTCACC
TpIFS-P_{TDH1}-F	ACTTCACTAAATTTACACACAAAACAAAATGTTGTTGGAAATTGCTGTTG
TpIFS-T_{PGK1}-R	TCTATCGATTTCAATTCAATTCAATTTAAGAAGACAACAATTTAGCAGCA
T_{PGK1}-F	ATTGAATTGAATTGAAATCGATAGATCA
T_{PGK1}-R	AAATTAAAACCGATTGACCAATATATGT
P_{PDC1}-T_{PGK1}-F	GACATATATTGGTCAATCGGTTTTAATTTTTATGTATGCTCTTCTGACTTTTCGT
P_{PDC1}-R	TTTGATTGATTTGACTGTGTTATTTT
GmCPR-P_{PDC1}-F	ACGCAAAATAACACAGTCAAATCAATCAAAATGCAGGACTCTGGTTCT
GmCPR-T_{TDH2}-R	AAATCATTAAAGTAACTTAAGGAGTTAAATTTACCAGACATCTCTCAAATATCTAC
LjCPR-P_{PDC1}-F	ACGCAAAATAACACAGTCAAATCAATCAAAATGGAGGAATCCTCTTCTATGAA
LjCPR-T_{TDH2}-R	AAATCATTAAAGTAACTTAAGGAGTTAAATTTACCAGACATCCCTCAAGTA
LjCPR-YZ-R	CCATAACAAATTCTCTGTTTTCCAAA
T_{TDH2}-F	ATTTAACTCCTTAAGTTACTTTAATGATTT
T_{TDH2}-R	ATGGACAGATATGCGATGAAAA
P_{CCW12}-T_{TDH2}-F	GCGTTTTCATCGCATATCTGTCCATTATTGGCGTCTGATTTCCGT
P_{CCW12}-T_{PGK1}-F	TATATTGGTCAATCGGTTTTAATTTTATTGGCGTCTGATTTCCGT
P_{CCW12}-R	TATTGATATAGTGTTTAAGCGAATGAC
GmHID-P_{CCW12}-F	TCATTCGCTTAAACACTATATCAATAATGGCTAAGGAGATCGTCAAGGAACTG

GmHID-T_{TEF1}-R	TATGCAACTAGAAAAGTCTTATCAATCTCCTTAAACCAAGAAAGAGGCCAATC
T_{TEF1}-YORWΔ17-R	TTTGAGAGCCCACTTTTGTTGGGGACGATTATAGCGCCGATCAAAGTATTT
YORWΔ17-DOWN-F	AATCGTCCCCAACAAAAGTGGG
YORWΔ17-DOWN-R	AAAGCTGGCTCCCCTTAGACAAATAC
YORWΔ17-YZ-F	CATATCTAATCACCCAATGTAGTTT
YORWΔ17-YZ-R	GCATTTCGTTCACTTACCAAAC
delta1-F	AAAAATCCACTATCGTCTATCAACT
delta1-loxp1-R	TGCAAGCTTGGCACTGGCCGTCGTTTTACACTCGAGGATATAGGAATCCTCAAAA
T_{PGK1}-delta2-R	TTAGGTATACAGAATATACTAGAAGTTCTCAAATTAAAACCGATTGACCAATATAT G
delta2-F	GAGAACTTCTAGTATATTCTGTATACCTAA
delta2-R	AACAGCTGATGAAGCAGGTG

Table S4. Plasmids used in this study.

Plasmids	Description	Source
pXP320	Multiple-copy plasmid with *loxp-HIS3-loxp*; Amp^R	[19]
pRS426	Multiple-copy plasmid with URA3-selectable marker; Amp^R	Novagen
pRS416	Single-copy plasmid with URA3-selectable marker; Amp^R	Novagen
pCDFDuet-1	pCDF ori with P_{T7}; Sm^R	Novagen
pCRE	pRS416 possessing P_{TDH3}-*Cre*- T_{ACS1}	This study
pMNG1	pRS426 possessing P_{SED1}-*EbCHS*-T_{ENO2}-P_{TEF1}-*Ha4CL*-T_{GPM1}-P_{TEF2}-*ErCHI*-T_{GPD}	This study
pMNG2	pRS426 possessing P_{SED1}-*SbCHS*-T_{ENO2}-P_{TEF1}-*Ha4CL*-T_{GPM1}-P_{TEF2}-*ErCHI*-T_{GPD}	This study
pMNG3	pRS426 possessing P_{SED1}-*PhCHS*-T_{ENO2}-P_{TEF1}-*Ha4CL*-T_{GPM1}-P_{TEF2}-*ErCHI*-T_{GPD}	This study
pMNG4	pRS426 possessing P_{SED1}-*HaCHS*-T_{ENO2}-P_{TEF1}-*Ha4CL*-T_{GPM1}-P_{TEF2}-*ErCHI*-T_{GPD}	This study
pMNG5	pRS426 possessing P_{SED1}-*AsCHS*-T_{ENO2}-P_{TEF1}-*Ha4CL*-T_{GPM1}-P_{TEF2}-*ErCHI*-T_{GPD}	This study
pMNG6	pRS426 possessing P_{SED1}-*GmCHS*-T_{ENO2}-P_{TEF1}-*Ha4CL*-T_{GPM1}-P_{TEF2}-*ErCHI*-T_{GPD}	This study
pMNG7	pRS426 possessing P_{SED1}-*PpCHS*-T_{ENO2}-P_{TEF1}-*Ha4CL*-T_{GPM1}-P_{TEF2}-*ErCHI*-T_{GPD}	This study
pMNG8	pRS426 possessing P_{SED1}-*MdCHS*-T_{ENO2}-P_{TEF1}-*Ha4CL*-T_{GPM1}-P_{TEF2}-*ErCHI*-T_{GPD}	This study
pMNG12	pRS426 possessing P_{PGK1}-*SbCHIL*-T_{GPD}	This study
pMNG13	pRS426 possessing P_{PGK1}-*PhCHIL*-T_{GPD}	This study
pMNG14	pRS426 possessing P_{PGK1}-*AtCHIL*-T_{GPD}	This study
pMNG15	pRS426 possessing P_{PGK1}-*MdCHIL*-T_{GPD}	This study
pMNG17	pRS416 possessing P_{TDH3}-*Ha4CL*-$(GGGGS)_3$-*EGFP*-T_{ACS1}	This study
pMNG18	pRS416 possessing P_{TDH3}-*MLS-Ha4CL*-$(GGGGS)_3$-*EGFP*-T_{ACS1}	This study
pMNG19	pRS416 possessing P_{TDH3}-*Ha4CL*-$(GGGGS)_3$-*EGFP-ePTS1*-T_{ACS1}	This study
pMNG20	pCDFDuet-1 possessing $PHA2_{up}$-*loxp-LEU2-loxp*-P_{TDH3}-*ARO4*^{K229L}-T_{ADH1}-P_{HXT7}-*ARO7*^{G141S}-T_{TEF1}-P_{HXK1}-*EcAROL*-T_{CYC1}-$PHA2_{down}$	This study

Supplementary File S1

The maps of key plasmids:

The skeleton of plasmids pMNG1-pMNG8 and pMNG12-pMNG15 is pRS426, and its frame sequence can be obtained at https://www.snapgene.com/resources/plasmid-files/?set=yeast_plasmids&plasmid=pRS426. All expression cassettes were inserted at the multiple cloning site (MCS). The difference of pMNG1-pMNG8 is the CHS sequences from different plant species. And the difference of pMNG12-pMNG15 is the CHIL sequences from different plant species. Therefore, maps of pMNG1 and pMNG12 were given as examples, respectively.

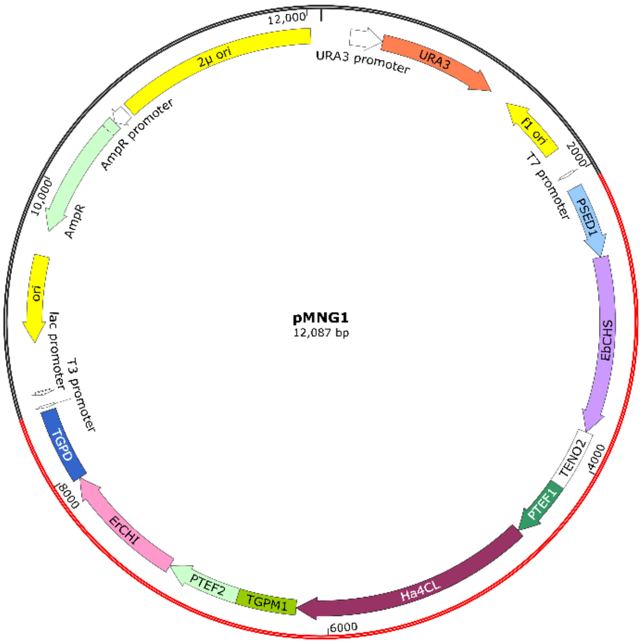

Figure S4. The map of pMNG1.

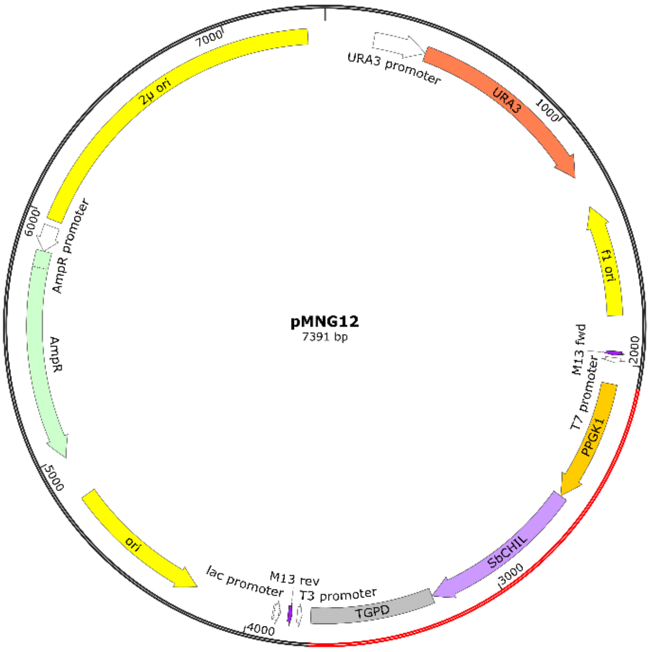

Figure S5. The map of pMNG12.

The skeleton of plasmids pMNG17-pMNG19 is pRS416 (https://www.snapgene.com/resources/plasmid-files/?set=yeast_plasmids&plasmid=pRS416). All expression cassettes were inserted at the multiple cloning site (MCS). The DNA sequence of (GGGGS)3 linker is (GGTGGTGGTGGTTCT)3. The maps of pMNG17-pMNG19 were given as follows.

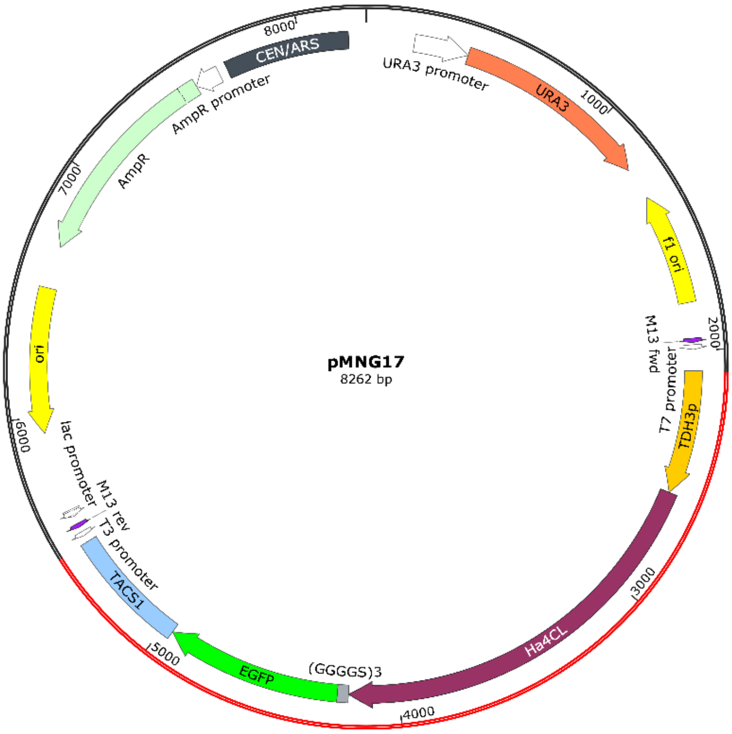

Figure S6. The map of pMNG17.

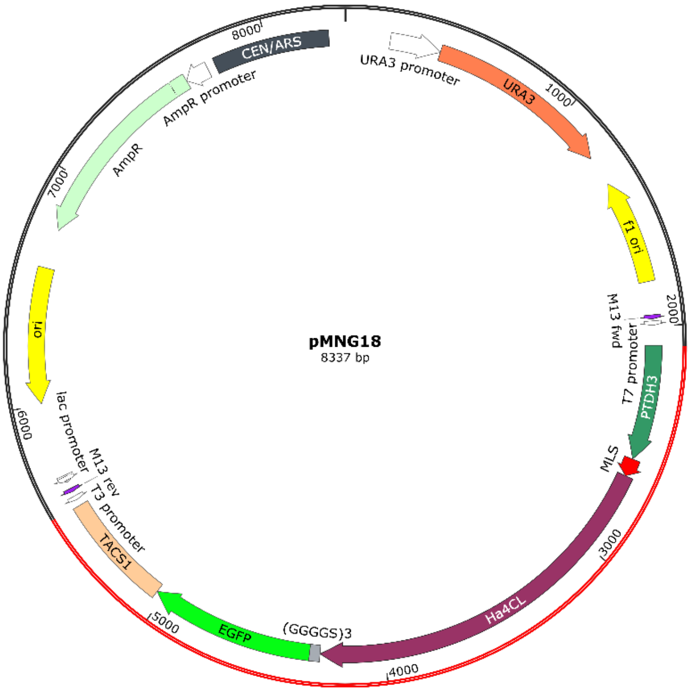

Figure S7. The map of pMNG18. MLS (atgctttcactacgtcaatctataagatttttcaagccagccacaagaactttgtgtagctctagatatctgcttcag).

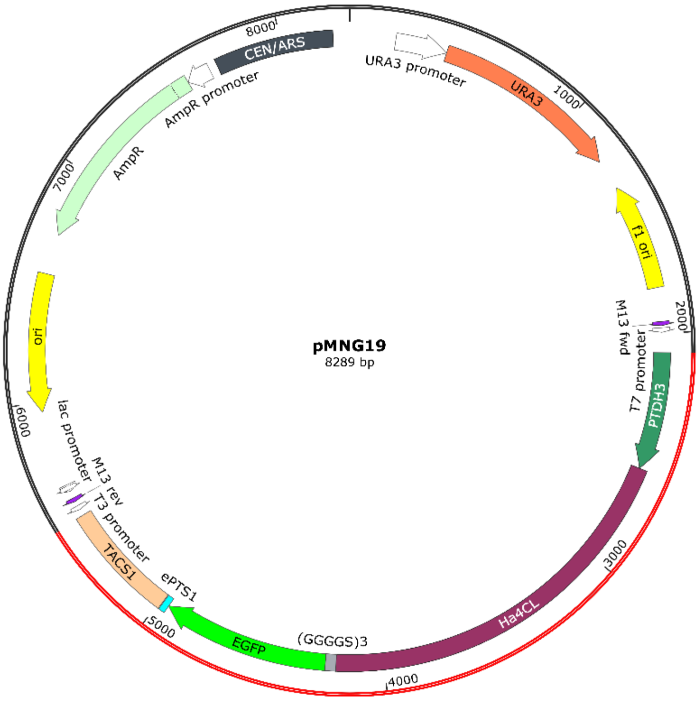

Figure S8. The map of pMNG19. ePTS1 (ttgggaagaggtagaagatccaaattg).

The skeleton of plasmid pMNG20 is pCDFDuet-1 (https://www.snapgene.com/resources/plasmid-files/?set=pet_and_duet_vectors_(novagen)&plasmid=pCDFDuet-1). All expression elements were inserted at the multiple cloning site 2 (MCS-2). The map of pMNG20 was given as follows.

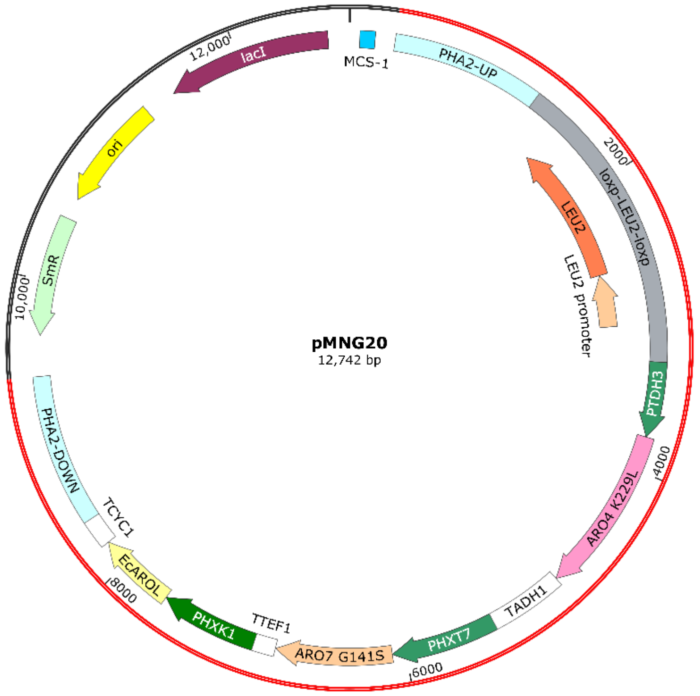

Figure S9. The map of pMNG20.

All gene sequences were given in TableS2 and Refs. 17, 18, and all promoters and terminators sequences were listed in TableS5.

Table S5. The sequences of promoters and terminators used in this study.

**Promoter**	**Sequence (5′-3′)**
P_{SED1}	GGACAAAAAGGCATATATCGCTAAAATTAGCCATCAGAACCGTTATTGTTATTATATTTTCATTACGAAAGAGGAGAGGG<br>CCCAGCGCGCCAGAGCACACACGGTCATTGATTACTTTATTTGGCTAAAGATCCATCCCTTCTCGATGTCATCTCTTTCCAT<br>TCTTGTGTATTTTTGATTGAAAATGATTTTTTGTCCACTAATTTCTAAAAATAAGACAAAAAGCCTTTAAGCAGTTTTTCAT<br>CCATTTTACTACGGTAAAATGAATTAGTACGGTATGGCTCCCAGTCGCATTATTTTTAGATTGGCCGTAGGGGCTGGGGTA<br>GAACTAGAGTAAGGAACATTGCTCTGCCCTCTTTTGAACTGTCATATAAATACCTGACCTATTTTATTCTCCATTATCGTATT<br>ATCTCACCTCTCTTTTTCTATTCTCTTGTAATTATTGATTTATAGTCGTAACTACAAAGACAAGCAAAATAAAATACGTTCG<br>CTCTATTAAG
P_{TEF1}	CCACACACCATAGCTTCAAAATGTTTCTACTCCTTTTTTACTCTTCCAGATTTTCTCGGACTCCGCGCATCGCCGTACCACT<br>TCAAAACACCCAAGCACAGCATACTAAATTTCCCCTCTTTCTTCCTCTAGGGTGTCGTTAATTACCCGTACTAAAGGTTTG<br>GAAAAGAAAAAAGAGACCGCCTCGTTTCTTTTTCTTCGTCGAAAAAGGCAATAAAAATTTTTATCACGTTTCTTTTTCTTG<br>AAAATTTTTTTTTTTGATTTTTTTCTCTTTCGATGACCTCCCATTGATATTTAAGTTAATAAACGGTCTTCAATTTCTCAAGTT<br>TCAGTTTCATTTTTCTTGTTCTATTACAACTTTTTTTACTTCTTGCTCATTAGAAAGAAAGCATAGCAATCTAATCTAAG
P_{TEF2}	GGGCGCCATAACCAAGGTATCTATAGACCGCCAATCAGCAAACTACCTCCGTACATTCATGTTGCACCCACACATTTATAC<br>ACCCAGACCGCGACAAATTACCCATAAGGTTGTTTGTGACGGCGTCGTACAAGAGAACGTGGGAACTTTTTAGGCTCAC<br>CAAAAAAGAAAGAAAAAATACGAGTTGCTGACAGAAGCCTCAAGAAAAAAAAAAATTCTTCTTCGACTATGCTGGAGGC<br>AGAGATGATCGAGCCGGTAGTTAACTATATATAGCTAAATTGGTTCCATCACCTTCTTTTCTGGTGTCGCTCCTTCTAGTGC<br>TATTTCTGGCTTTTCCTATTTTTTTTTTTCCATTTTTCTTTCTCTCTTTCTAATATATAAATTCTCTTGCATTTTCTATTTTTCTCTC<br>TATCTATTCTACTTGTTTATTCCCTTCAAGGTTTTTTTTTAAGGAGTACTTGTTTTTAGAATATACGGTCAACGAACTATAATT<br>AACTAAAC
P_{PGK1}	TGTTTGCAAAAAGAACAAAACTGAAAAAACCCAGACACGCTCGACTTCCTGTCTTCCTATTGATTGCAGCTTCCAATTTC<br>GTCACACAACAAGGTCCTAGCGACGGCTCACAGGTTTTGTAACAAGCAATCGAAGGTTCTGGAATGGCGGGAAAGGGT<br>TTAGTACCACATGCTATGATGCCCACTGTGATCTCCAGAGCAAAGTTCGTTCGATCGTACTGTTACTCTCTCTCTTTCAAAC<br>AGAATTGTCCGAATCGTGTGACAACAACAGCCTGTTCTCACACACTCTTTTCTTCTAACCAAGGGGGTGGTTTAGTTTAGT<br>AGAACCTCGTGAAACTTACATTTACATATATATAAACTTGCATAAATTGGTCAATGCAAGAAATACATATTTGGTCTTTTCT<br>AATTCGTAGTTTTTCAAGTTCTTAGATGCTTTCTTTTTCTCTTTTTTACAGATCATCAAGGAAGTAATTATCTACTTTTTACAA<br>CAAATATAAAACA
P_{TDH3}	ACAGTTTATTCCTGGCATCCACTAAATATAATGGAGCCCGCTTTTTAAGCTGGCATCCAGAAAAAAAAAGAATCCCAGCA<br>CCAAAATATTGTTTTCTTCACCAACCATCAGTTCATAGGTCCATTCTCTTAGCGCAACTACAGAGAACAGGGGCACAAAC<br>AGGCAAAAAACGGGCACAACCTCAATGGAGTGATGCAACCTGCCTGGAGTAAATGATGACACAAGGCAATTGACCCAC<br>GCATGTATCTATCTCATTTTCTTACACCTTCTATTACCTTCTGCTCTCTCTGATTTGGAAAAAGCTGAAAAAAAAGGTTGAA<br>ACCAGTTCCCTGAAATTATTCCCCTACTTGACTAATAAGTATATAAAGACGGTAGGTATTGATTGTAATTCTGTAAATCTATT<br>TCTTAAACTTCTTAAATTCTACTTTTATAGTTAGTCTTTTTTTTAGTTTTAAAACACCAAGAACTTAGTTTCGAATAAACACA<br>CATAAACAAACAAA
P_{HXT7}	TCTAGTTTCTGCCTTAAACAAAGCCGCAGCCAGAGCCGTTTTTCCGCCATATTTATCCAGGATTGTTCCATACGGCTCCGT<br>CAGAGGCTGCTACGGGATGTTTTTTTTTTACCCCGTGGAAATGAGGGGTATGCAGGAATTTGTGCGGGGTAGGAAATCTTT<br>TTTTTTTTTAGGAGGAACAACTGGTGGAAGAATGCCCACACTTCTCAGAAATGCATGCAGTGGCAGCACGCTAATTCGAA<br>AAAATTCTCCAGAAAGGCAACGCAAAATTTTTTTTCCAGGGAATAAACTTTTTATGACCCACTACTTCTCGTAGGAACAA<br>TTTCGGGCCCCTGCGTGTTCTTCTGAGGTTCATCTTTTACATTTGCTTCTGCTGGATAATTTTCAGAGGCAACAAGGAAAA<br>ATTAGATGGCAAAAAGTCGTCTTTCAAGGAAAAATCCCCACCATCTTTCGAGATCCCCTGTAACTTATTGGCAACTGAAA<br>GAATGAAAAGGAGGAAAATACAAAATATACTAGAACTGAAAAAAAAAAAGTATAAATAGAGACGATATATGCCAATAC<br>TTCACAATGTTCGAATCTATTCTTCATTTGCAGCTATTGTAAAATAATAAAACATCAAGAACAAACAAGCTCAACTTGTCT

TTTCTAAGAACAAAGAATAAACACAAAAACAAAAAGTTTTTTTAATTTTAATCAAAAA

P_{HXK1}
TTTAGACGCTTTTAAGGAGTACGTGTTCCTGTCGTTTATCCACTAAGTATACTGGTTGCCCCTGGCCAGATCTCAGTATAGC
AGTGACGCGTGGGTTTCAGGAAGAATGGCAGTCCCCTTTTGTTTTTCCGCATTGTGTGAGCTTCTTATGCCCCTGAACCCC
ACTATTCTGCCCCTTTGAAACTCCCGCACGTGTGCCCCGTTTGTTGGAAGATAGCGAAATACCTTACTGGAGCAACCAGG
AAAAATACTCTGGTTGCAAAAACCAACAAAAGAAAAAATGGAAGACCTAAGAACTATGCATTTTTTTTTAAGGGGTTGA
TGAAAAGAAAAAGTTTCTTTCTCCCCCCGGATTTTGGTACCTTAGGACCGTTGAGAGGAATAGTAACAAGTGAACGCAA
CAAAGATTGTTCTCAACTGCTTCTGTTTCCTCCTTTTCTTTAAAGAGGAATATTTCGTATATAAGCAATCGGTTTCACTTCCT
TGGGAATATTCTACCGTTCCTTCATCTTGTATTCTTCTCTTTCTCTTAGCGCAATATATAGCAGAAGAGCAATAAGAAACAA
TTGTGGCTTGCAATACTCAATTAGAATTCTTTTCTTTTAATCAAACTCACCCAAACAACTCAATTAGAATACTGAAAAAAT
AAG

P_{TDH1}
GGAATAGGATATGCGACGAAGACGCTTCTGCTTAGTAACCACACCACATTTTCAGGGGGTCGATCTGCTTGCTTCCTTTAC
TGTCACGAGCGGCCCATAATCGCGCTTTTTTTTTAAAAGGCGCGAGACAGCAAACAGGAAGCTCGGGTTTCAACCTTCG
GAGTGGTCGCAGATCTGGAGACTGGATCTTTACAATACAGTAAGGCAAGCCACCATCTGCTTCTTAGGTGCATGCGACGG
TATCCACGTGCAGAACAACATAGTCTGAAGAAGGGGGGGAGGAGCATGTTCATTCTCTGTAGCAGTAAGAGCTTGGTGA
TAATGACCAAAACTGGAGTCTCGAAATCATATAAATAGACAATATATTTTCACACAATGAGATTTGTAGTACAGTTCTATT
CTCTCTCTTGCATAAATAAGAAATTCATCAAGAACTTGGTTTGATATTTCACCAACACACACAAAAAACAGTACTTCACT
AAATTTACACACAAAACAAA

P_{PDC1}
TTATGTATGCTCTTCTGACTTTTCGTGTGATGAGGCTCGTGGAAAAAATGAATAATTTATGAATTTGAGAACAATTTTGTGT
TGTTACGGTATTTTACTATGGAATAATCAATCAATTGAGGATTTTATGCAAATATCGTTTGAATATTTTTCCGACCCTTTGAG
TACTTTTCTTCATAATTGCATAATATTGTCCGCTGCCCCTTTTTCTGTTAGACGGTGTCTTGATCTACTTGCTATCGTTCAACA
CCACCTTATTTTCTAACTATTTTTTTTTTAGCTCATTTGAATCAGCTTATGGTGATGGCACATTTTTGCATAAACCTAGCTGTC
CTCGTTGAACATAGGAAAAAAAAAATATATAAACAAGGCTCTTTCACTCTCCTTGCAATCAGATTTGGGTTTGTTCCCTTTA
TTTTCATATTTCTTGTCATATTCCTTTCTCAATTATTATTTTCTACTCATAACCTCACGCAAAATAACACAGTCAAATCAATC
AAA

P_{CCW12}
TATTGGCGTCTGATTTCCGTTTTGGGAATCCTTTGCCGCGCGCCCCTCTCAAAACTCCGCACAAGTCCCAGAAAGCGGGA
AAGAAATAAAACGCCACCAAAAAAAAAAAAAATAAAAGCCAATCCTCGAAGCGTGGGTGGTAGGCCCTGGATTATCCCG
TACAAGTATTTCTCAGGAGTAAAAAAACCGTTTGTTTTGGAATTCCCCATTTCGCGGCCACCTACGCCGCTATCTTTGCAA
CAACTATCTGCGATAACTCAGCAAATTTTGCATATTCGTGTTGCAGTATTGCGATAATGGGAGTCTTACTTCCAACATAAC
GGCAGAAAGAAATGTGAGAAAATTTTGCATCCTTTGCCTCCGTTCAAGTATATAAAGTCGGCATGCTTGATAATCTTTCTT
TCCATCCTACATTGTTCTAATTATTCTTATTCTCCTTTATTCTTTCCTAACATACCAAGAAATTAATCTTCTGTCATTCGCTTA
AACACTATATCAATA

Terminator

T_{ENO2}
AGTGCTTTTAACTAAGAATTATTAGTCTTTTCTGCTTATTTTTTCATCATAGTTTAGAACACTTTATATTAACGAATAGTTTAT
GAATCTATTTAGGTTTAAAAATTGATACAGTTTTATAAGTTACTTTTTCAAAGACTCGTGCTGTCTATTGCATAATGCACTG
GAAGGGGAAAAAAAAGGTGCACACGCGTGGCTTTTTCTTGAATTTGCAGTTTGAAAAATAACTACATGGATGATAAGAA
AACATGGAGTACAGTCACTTTGAGAACCTTCAATCAGCTGGTAACGTCTTCGTTAATTGGATACTCAAAAAAGATGGATA
GCATGAATCACAAGATGGAAGGAAATGCGGGCCACGACCACAGTGATATGCATATGGGAGATGGAGATGATACCT

T_{GPM1}
GTCTGAAGAATGAATGATTTGATGATTTCTTTTTCCCTCCATTTTTCTTACTGAATATATCAATGATATAGACTTGTATAGTTT
ATTATTTCAAATTAAGTAGCTATATATAGTCAAGATAACGTTTGTTTGACACGATTACATTATTCGTCGACATCTTTTTTCAG
CCTGTCGTGGTAGCAATTTGAGGAGTATTATTAATTGAATAGGTTCATTTTGCGCTCGCATAAACAGTTTTCGTCAGGGAC
AGTATGTTGGAATGAGTGGTAATTAATGGTGACATGACATGTTATAGCAATAACCTTGATGTTTACATCGTAGTTTAATGTA
CACCCCGCGAATTCGTTCAAGTAGGAGTGCACCAATTGCAAAGGGAAAAGCTGAATGGGCAGTTCGAATA

T_{GPD}
GTGAATTTACTTTAAATCTTGCATTTAAATAAATTTTCTTTTTATAGCTTTATGACTTAGTTTCAATTTATATACTATTTTAATG

	ACATTTTCGATTCATTGATTGAAAGCTTTGTGTTTTTTCTTGATGCGCTATTGCATTGTTCTTGTCTTTTTCGCCACATGTAAT<br>ATCTGTAGTAGATACCTGATACATTGTGGATGCTGAGTGAAATTTTAGTTAATAATGGAGGCGCTCTTAATAATTTTGGGGA<br>TATTGGCTTTTTTTTTTAAAGTTTACAAATGAATTTTTTCCGCCAGGATAACGATTCTGAAGTTACTCTTAGCGTTCCTATCG<br>GTACAGCCATCAAATCATGCCTATAAATCATGCCTATATTTGCGTGCAGTCAGTATCATCTACATGAAAAAAACTCCCGCA<br>ATTTCTTATAGAATACGTTGAAAATTAAATGTACGCGCCAAGATAAGATAACATATATCTAGATGCAGTAATATACACAGA<br>TTCC
T_{ACS1}	TGATGATTTCTTTCCTTTTTATATTGACGACTTTTTTTTTTTCGTGTGTTTTTGTTCTCTTATAACCGAGCTGCTTACTTATTATT<br>ATTTCACCTTCTCTTTTTATTTATACTTATAATTATTTATTCTTTACATACTGTTACAAGAAACTCTTTTCTACATTAATTGCAT<br>AAAGTGTCAATCAGCACATCCTCTACATCGCTATCAACAACAAATTTGACAAACCTGCCTATATCTTCAGGAACGACTGC<br>TGCATCGCTACCACCACTACTTGTGAAGTCCCTGGAGTTCAATATGCACTGAAATTTACCTAGCCGTCTTACACATGACCA<br>TAATCCATCCATGCTATCGCAATATATGATTTTGTGTTCGTTTTTCGTCTTGCGAAAGGCATCCCCAATGGCTTGTTTCATTG<br>ATCCATCAGTGTGGCTCGTAGGTACCAGCAAAACCACTTCATCAGCGGCGTACTCCTCCCACTTTATGGGCAGTCCTTGTA<br>TCGA
T_{ADH1}	GCGAATTTCTTATGATTTATGATTTTTATTATTAAATAAGTTATAAAAAAAATAAGTGTATACAAATTTTAAAGTGACTCTTA<br>GGTTTTAAAACGAAAATTCTTATTCTTGAGTAACTCTTTCCTGTAGGTCAGGTTGCTTTCTCAGGTATAGCATGAGGTCGCT<br>CTTATTGACCACACCTCTACCGGCATGCCGAGCAAATGCCTGCAAATCGCTCCCCATTTCACCCAATTGTAGATATGCTAA<br>CTCCAGCAATGAGTTGATGAATCTCGGTGTGTATTTTATGTCCTCAGAGGACAACACCTGTTGTAATCGTTCTTCCACACG<br>GATCCACAGCCTAGCCTTCAGTTGGGCTCTATCTTCATCGTCATTCATTGCATCTACTAGCCCCTTACCTGAGCTTCAAGAC<br>GTTATATCGCTTTTATGTATCATGATCTTATCTTGAGATATGAATACATAAATATATTTACTCAAGTGTATACGTGCATGCTTT<br>TTTTACG
T_{TEF1}	AGGAGATTGATAAGACTTTTCTAGTTGCATATCTTTTATATTTAAATCTTATCTATTAGTTAATTTTTTGTAATTTATCCTTATA<br>TATAGTCTGGTTATTCTAAAATATCATTTCAGTATCTAAAAATTCCCCTCTTTTTTCAGT
T_{CYC1}	ATCCGCTCTAACCGAAAAGGAAGGAGTTAGACAACCTGAAGTCTAGGTCCCTATTTATTTTTTTATAGTTATGTTAGTATTA<br>AGAACGTTATTTATATTTCAAATTTTTCTTTTTTTTCTGTACAGACGCGTGTACGCATGTAACATTATACTGAAAACCTTGCT<br>TGAGAAGGTTTTGGGACGCTCGAAG
T_{PGK1}	ATTGAATTGAATTGAAATCGATAGATCAATTTTTTTCTTTTCTCTTTCCCCATCCTTTACGCTAAAATAATAGTTTATTTTATT<br>TTTTGAATATTTTTTATTTATATACGTATATATAGACTATTATTTATCTTTTAATGATTATTAAGATTTTTATTAAAAAAAAATTC<br>GCTCCTCTTTTAATGCCTTTATGCAGTTTTTTTTTCCCATTCGATATTTCTATGTTCGGGTTCAGCGTATTTTAAGTTTAATAAC<br>TCGAAAATTCTGCGTTCGTTAAAGCTTTCGAGAAGGATATTATTTCGAAATAAACCGTGTTGTGTAAGCTTGAAGCCTTTT<br>TGCGCTGCCAATATTCTTATCCATCTATTGTACTCTTTAGATCCAGTATAGTGTATTCTTCCTGCTCCAAGCTCATCCCACTT<br>GCAACAAAAAAAGTCTAATCTTCTGCAATAATTTCCATCCTTGGCATTCAGAGACATATATTGGTCAATCGGTTTTAATTT
T_{TDH2}	ATTTAACTCCTTAAGTTACTTTAATGATTTAGTTTTTATTATTAATAATTCATGCTCATGACATCTCATATACACGTTTATAAA<br>ACTTAAATAGATTGAAAATGTATTAAAGATTCCTCAGGGATTCGATTTTTTTGGAAGTTTTTGTTTTTTTTTCCTTGAGATGC<br>TGTAGTATTTGGGAACAATTATACAATCGAAAGATATATGCTTACATTCGACCGTTTTAGCCGTGATCATTATCCTATAGTA<br>ACATAACCTGAAGCATAACTGACACTACTATCATCAATACTTGTCACATGAGAACTCTGTGAATAATTAGGCCACTGAAA<br>TTTGATGCCTGAAGGACCGGCATCACGGATTTTCGATAAAGCACTTAGTATCACACTAATTGGCTTTTCGCCGCATATGGT<br>GTTTCCGGTGATTTCCAAGTATTGTTTCCAAGCATCGTACCTTTCACCATTTGGAGTATCACTTAGCGTTTTCATCGCATATC<br>TGTCCAT
